# Metabolic reprogramming of abscisic acid-producing strain *Botrytis cinerea* TB-31 toward terpenoid biosynthesis using a CRISPR/Cas9 ribonucleoprotein system

**DOI:** 10.1016/j.synbio.2025.12.002

**Published:** 2025-12-31

**Authors:** Xiao-Nan Hou, Dan Shu, Tian-Fu Li, Qi Yang, Zhe-Min Li, Di Luo, Jie Yang, Zhi-Ying Yan, Hong Tan

**Affiliations:** a Agricultural Microbial Agents Key Laboratory of Sichuan Province, Chengdu Institute of Biology, Chinese Academy of Sciences, Chengdu, 610041, PR China; b University of Chinese Academy of Sciences, Beijing, 100049, PR China

**Keywords:** Filamentous fungi, *Botrytis cinerea* TB-31, Abscisic acid (ABA), CRISPR-Cas9 ribonucleoprotein complexes, Terpenoid biosynthesis

## Abstract

Compared with conventional microbial hosts, filamentous fungi have distinct advantages for the industrial-scale biosynthesis of high-value chemical compounds. However, current research on strain engineering and fermentation optimization strategies for synthetic biology applications is limited in filamentous fungi, especially in industrial production strains. In this study, we established a CRISPR/Cas9-based gene editing system in *Botrytis cinerea* strain TB-31, an important filamentous fungal platform for the study of the biosynthesis and regulation of the sesquiterpenoid abscisic acid (ABA). This system enables efficient single- and multigene knockout, large-fragment deletion, and heterologous protein expression. Among the engineered mutant strains, the △*bcaba1234* strain with complete ablation of the ABA biosynthetic gene cluster (BGC) demonstrated significant metabolic flux rewiring, redirecting cellular resources toward terpenoid precursor biosynthesis; this metabolic reprogramming proves pivotal for high-value terpenoid biosynthesis. This study not only establishes an efficient genome editing tool for the ABA-producing strain *B. cinerea* TB-31 but also provides a foundation for its development as a new potential terpenoid-producing chassis strain.

## 1 Introduction

Filamentous fungi are eukaryotic microorganisms known for their characteristic hyphal structures, making them a multicellular morphological taxon within the kingdom Fungi. These fungi can produce a wide range of secondary metabolites, including terpenoids, polyketides, and nonribosomal peptides [1], among others. Terpenoids, a diverse class of natural products derived from isoprene (C5) units, including a wide range of isoprenoid derivatives, have gained significant industrial importance in various sectors, such as the pharmaceutical, nutraceutical, flavor, and bioenergy sectors [2]. Due to the increasing demand for these valuable compounds, there has been a push for the development of advanced bioproduction strategies that utilize sustainable approaches to increase terpenoid yields.

Terpenoid biosynthesis in filamentous fungi primarily occurs through the mevalonate (MVA) pathway, in which isopentenyl diphosphate (IPP) and dimethylallyl diphosphate (DMAPP) serve as fundamental building blocks [3]. Prenyltransferases sequentially condense these C5 precursors to form linear isoprenoid diphosphates of varying chain lengths, including geranyl diphosphate (GPP, C10), farnesyl diphosphate (FPP, C15), and geranylgeranyl diphosphate (GGPP, C20) [4]. Subsequent enzymatic modifications, such as cyclization or rearrangement catalyzed by terpene synthases, followed by oxidative transformations mediated by cytochrome P450s and dehydrogenases, yield structurally diverse terpenoids with distinct biological activities [5]. Some microbial hosts, including filamentous fungi such as *Aspergillus oryzae*, *Ustilago maydis* and *Aspergillus nidulans*, enable the reconstruction of well-characterized terpenoid biosynthetic pathways through recent advances in synthetic biology, making heterologous production of these compounds achievable via metabolic engineering strategies [[6], [7], [8], [9]]. Despite their considerable biosynthetic potential, filamentous fungi have yet to be fully developed as efficient terpene production platforms. The multinucleated nature of filamentous fungi introduces inherent complexities, resulting in current limitations such as inefficient gene editing and a lack of robust metabolic engineering methods [10].

With its fully sequenced and well-annotated genome, *Botrytis cinerea* is an ideal model filamentous fungus for fundamental research on plant pathology and cell biology [11]. A genomic analysis of *B. cinerea* revealed 24 secondary metabolite biosynthetic gene clusters (BGCs), including 10 terpene synthases (TSs), 7 polyketide synthases (PKSs), 3 nonribosomal peptide synthetases (NRPSs), and 4 PKS-NRPS hybrid enzymes [12,13], indicating the potential of this species for bioactive metabolite discovery. In 1982, *B. cinerea* was found to synthesize abscisic acid (ABA) [14]. ABA, a biologically active sesquiterpenoid, is widely recognized as a “stress” hormone due to its critical role in enhancing plant resistance to abiotic and biotic stresses [15]. Our research group subjected the wild-type *B. cinerea* strain TBC-6 to multiple rounds of mutagenesis and screening over a 20-year period, resulting in the development of mutant strains TB-31 and TB-3-H8 with significantly increased ABA yields [16]. Subsequent strain improvement led to the generation of TBC-A, a robust industrial production strain [17]. Among these strains, the TB-31 strain serves as the chassis organism for investigations into ABA biosynthesis and regulatory mechanisms. Through comprehensive molecular characterization, the complete ABA biosynthetic pathway in *B. cinerea* TB-31 has been elucidated, including the identification of key regulatory elements that govern ABA biosynthesis [[18], [19], [20], [21], [22], [23], [24]]. In *B. cinerea*, ABA biosynthesis occurs through the MVA pathway and involves a dedicated gene cluster comprising four key enzymes, *bcaba1* and *bcaba2*, which encode cytochrome P450 monooxygenases responsible for C-1′ and C-4′ hydroxylation [25]; *bcaba3*, encoding a sesquiterpene synthase catalytic subunit [[26], [27], [28], [29]]; and *bcaba4*, encoding a short-chain dehydrogenase/reductase (SDR) that mediates C-4′ oxidation. Transcriptomic analyses conducted in our laboratory have demonstrated significant upregulation of terpenoid biosynthetic genes and enhanced metabolic flux in the ABA-hyperproducing strain [20]. Furthermore, we systematically optimized the cultivation period, growth conditions, fermentation parameters, and product separation/purification processes for *B. cinerea*, demonstrating its proven industrial applicability. While these findings establish *B. cinerea* TB-31 as a promising microbial platform, several research avenues remain to be explored, including (1) systematic strain optimization with an efficient gene editing system and (2) metabolic engineering for enhanced terpenoid production and the biosynthesis of alternative high-value terpenoids beyond ABA.

CRISPR/Cas9 is a powerful tool for editing genomes and is typically used to modify secondary metabolite BGCs in filamentous fungi for research purposes and to improve product yields [30]. However, the editing efficiency of most filamentous fungi, particularly nonmodel species, is relatively low [31]; this is mainly due to the thick cell walls of these fungi, which make it difficult for exogenous DNA to enter the cells and limit the delivery efficiency of the CRISPR/Cas9 system. Additionally, the low efficiency of homologous recombination (HR) in filamentous fungi hinders the precise insertion of genes [32]. Furthermore, the multinucleated or multicellular nature of filamentous fungi can result in uneven gene editing efficiency. For example, in *B. cinerea*, the multinucleate nature of cells can cause edited strains to revert to their unedited state. In 2020, a ribonucleoprotein (RNP)-based CRISPR/Cas9 genome editing platform was successfully established in the important necrotrophic plant pathogen *B. cinerea* B05.10 to study virulence-related genes [33]. However, due to the inherent characteristics of industrial strains, such as aneuploidy, polyploidy, frequent chromosomal rearrangements, limited use of auxotrophic markers, and potential regulatory constraints associated with antibiotic resistance markers in commercial applications [34], CRISPR/Cas9 technology has not yet been successfully applied to ABA-producing strains.

In this study, three different editing systems for CRISPR/Cas9 were tested. Ultimately, an RNP-based CRISPR/Cas9 platform was developed and optimized specifically for *B. cinerea* strain TB-31. This platform effectively minimizes the impact of the Cas9 protein on *B. cinerea*. Using this system, we successfully edited single or multiple genes within the ABA BGC, resulting in a series of ABA precursors. We also constructed a potential terpenoid-producing chassis strain, △*bcaba1234*, by deleting *bcaba1*, *bcaba2*, *bcaba3*, and *bcaba4* from TB-31. Compared with the parental strain TB-31, the △*bcaba1234* strain presented an enhanced MVA pathway, as confirmed by transcriptional and metabolic analyses. Our CRISPR-mediated targeted integration platform enables efficient red fluorescent protein (mCherry) expression in △*bcaba1234*. Therefore, this method can be effectively employed for both the overexpression of key metabolic pathway genes and the construction of terpenoid-producing strains. This study successfully demonstrates the application of an RNP-based CRISPR/Cas9 system in the ABA-producing strain *B. cinerea* TB-31; it expands the genetic editing toolkit, broadens its biotechnological utility, and highlights its potential as a platform for large-scale terpenoid biosynthesis.

## 2 Materials and methods

### 2.1 Strains

*B. cinerea* TB-31, which was derived from wild-type strain *B. cinerea* TBC-6 isolated by our laboratory and conserved in our laboratory, was cultured on potato glucose agar (PDA) media at 26 °C for use. For transcriptome and metabolome analysis, the strains were cultured in the liquid fermentation medium at 25 °C, 200 rpm.

### 2.2 Plasmid construction

Various types of nucleic acid fragments were amplified using their corresponding primers (Table S1). Plasmids were constructed for the purpose of fluorescence microscopy, gene editing and protein expression. All constructs utilized in this study were verified through DNA sequencing. The operations are as follows:

The codon-optimized *Cas9* from *Streptococcus pyogenes* was obtained from the plasmid p414-TEF1p-*Cas9-CYC1t* (Addgene number: #43802) and used for expression in *B*. *cinerea* TB-31. The fragment Cas9-EGFP-4NLSs representing Cas9 fused with enhanced green fluorescent protein (EGFP) and four tandem nuclear localization signals (4NLSs) was obtained by overlap polymerase chain reaction (PCR) and cloned into the vector pCBh1 previously constructed by our laboratory [35] to produce pCBh1-*Cas9*-*EGFP*-*4NLSs*. The Cas9-4NLSs coding sequence was cloned into pCBh1 and pCBg418 to yield the pCBh1-*Cas9-4NLSs* or pCBg418-*Cas9-4NLSs* plasmids.

The codon-optimized *Cas9* fused with four NLSs was amplified with primers *Cas9*-F/R and then cloned into the vector pET28a to produce pET28a-*Cas9*-*4NLSs*. The fragment Cas9-4NLSs was amplified with primers *PpkiA*-*Cas9*-F and *Cas9*-*TglaA*-R using pET28a-*Cas9*-*4NLSs* as template, and cloned into the *Nhe*I and *Pac*I sites of vector Anep8-Cas9 (Addgene number: #117169) to generate pAnep8-*Cas9*-*4NLSs*-*AMA1*.

Construction of sgRNA-containing plasmid pJET1.2-pU6-sgRNA-*laeA* was performed as follows: First, the 20-nt target sequence of *bclaeA* was designed and synthesized. This sequence, along with the gRNA scaffold from p426-SNR52p-gRNA (Addgene number: #43803) and the *B. cinerea* U6 promoter, was assembled into a PU6-20 nt-gRNA scaffold construct. Finally, the resulting fragment was cloned into the blunt-end cloning vector pJET1.2 (Addgene number: #173156) for amplification.

The plasmid pTEL-*hyg*, which contains a pair of telomeres and provides transiently hygromycin resistance, was kindly provided by Prof. Matthias Hahn (University of Kaiserslautern, Department of Biology, Kaiserslautern, Germany).

The fragments used as donor DNA were constructed through a two-step process. Initially, PCR amplification was performed to obtain three distinct DNA fragments: the 5′ homologous arm, the *G418* resistance gene expression cassette (PanTrpC::G418), and the 3′ homologous arm. Then these fragments were subsequently assembled via overlap extension PCR and subsequently introduced into protoplasts for transformation. For heterologous expression of the mCherry protein, the PgpdA promoter-driven mCherry expression cassette (PgpdA:mCherry) was obtained from plasmid pCAMBIA1303 (MiaoLing Bio, Cat#P46327); homologous arms adjacent to the G418 resistance gene were then added via overlap PCR to generate the donor DNA.

### 2.3 *Agrobacterium tumefaciens* EHA105-mediated transformation (ATMT)

ATMT was carried out with minor modifications [36,37]. The recombinant plasmid pCBh1-*Cas9*-*EGFP*-*4NLSs* or other indicated plasmid was transfected into *A. tumefaciens* EHA105 and incubated at 28 °C and 220 rpm until the OD_600_ reached 0.35–0.45. A mixture of 100 μL of conidia (1 × 10^7^) from *B. cinerea* TB-31 or its modified strains and 100 μL of bacteria was then spread onto solid medium with a nitrocellulose (NC) membrane and incubated for 2 days. The membrane was aseptically transferred onto PDA solid medium supplemented with both hygromycin B (50 μg/mL; Sigma-Aldrich, St. Louis, MO, USA) and 200 μM cefotaxim, followed by incubation at 28 °C for 3–7 days until transformant colonies emerged. For purification of putative positive transformants, single conidial isolates were successively subcultured for at least three generations on PDA plates containing the respective selection antibiotics.

### 2.4 Fluorescence microscopy

The plasmid pCBh1-*Cas9-EGFP-4NLSs* was transformed into *B. cinerea* TB-31 using the ATMT method. Transformants were selected by growing them on PDA plates containing 50 μg/mL of hygromycin B and confirmed by PCR using the primer pair of EGFP-F/4NLSs-R. The single fungal colonies of the transformants were cultured on PDA plates added with sterile slide. After 72 h of incubation, hyphal samples were mounted in 10 μL antifade medium containing DAPI (1.5 μg/mL) and analyzed for nuclear fluorescence using a Leica DMi8 inverted fluorescence microscope equipped with a 63 × oil-immersion objective. For red fluorescence mCherry observation, the samples were examined under identical conditions but with a 40 × objective.

### 2.5 Cas9 protein expression and purification

The plasmid pET28a-*Cas9*-*4NLSs* was transformed into chemically competent *E. coli* Tssetta (DE3) cells (ESC-E04, Tsingke Biotech Co., Ltd., Beijing, China). When the optical density at 600 nm reached 0.6, the final concentration of 0.2 mM isopropyl β-d-1-thiogalactopyranoside (IPTG) was added to the medium. After the overnight incubation, the cells were collected and disrupted in an extraction buffer (20 mM HEPES, 500 mM NaCl, 1 mM DTT, pH 7.5) using ultrasonication for 10 min. Then the cell homogenate was centrifuged at 4 °C for 30 min. The supernatant was loaded onto a HisTrap Ni-NTA column and incubated on a rotator at 4 °C for 1 h. Unbounded proteins were washed away with 20 mM imidazole in the extraction buffer. The bound protein was eluted with 5 column volumes of elution buffer (20 mM HEPES, 200 mM imidazole, 500 mM NaCl, pH 8.0, 1 mM DTT, 2 mM MgCl_2_, 5 % glycerol). The protein was then concentrated and buffer-exchanged using a 10 kDa Vivaspin column. After measuring the protein concentration using the bicinchoninic acid (BCA) method, the final protein was frozen in liquid nitrogen and stored at −80 °C until use.

### 2.6 Synthesis of sgRNA and RNP formation

The sgRNAs used in this study were synthesized by *in vitro* transcription from DNA templates, as previously described with minor modifications [33]. First, the conserved domains of the target genes were analyzed on the National Center for Biotechnology Information (NCBI) website and used to design and select the highly specificity sgRNAs. The target site of 20 nt was then synthesized as oligonucleotides with a T7 promoter and annealed with a constant oligonucleotide to form double-stranded DNA templates. The sgRNAs were transcribed using the T7 High Yield RNA Transcription Kit (Nanjing Vazyme Biotech Co., Ltd., Nanjing, China), and then purified using the RNA Clean & Concentrator Kit (Zymo Research Corp., Irvine, California, USA). To form the RNPs, the purified Cas9 protein and matured sgRNAs were added to an incubation buffer (20 mM HEPES, pH 7.5, 100 mM KCl, 5 % glycerol, 1 mM DTT, 0.5 mM EDTA, 2 mM MgCl_2_) in a 3:2 ratio and incubated at 37 °C for 30 min.

### 2.7 *In vitro* cleavage assay for different sgRNAs

Several sgRNAs (4 μg) targeting the same gene, such as *bclaeA*, were separately incubated with purified 6 μg of Cas9 protein in cleavage buffer (20 mM HEPES, pH 7.5, 100 mM KCl, 5 % glycerol, 1 mM DTT, 0.5 mM EDTA, 2 mM MgCl_2_) at 37 °C for 15 min. Then, the fragment of the targeted gene's CDS was added to the reaction system for 1 h at 37 °C. To terminate the reaction, 1 mg/mL protease K, 10 mM CaCl_2_, and 1 % sodium dodecyl sulfate (SDS) were added and incubated in 55 °C for 1 h, and the supernatant was then loaded into bands using 2 % electrophoresis after centrifugation.

### 2.8 Transformation of *B*. *cinerea*

The transformation of *B*. *cinerea* protoplasts was performed using a previously published protocol with some modifications [38]. Firstly, 2 × 10^7^ mature spores were spread on a PDA plate covered with cellophane to form protoplasts. The germinated mycelia were harvested after 72 h and washed twice with KC buffer (0.6 M KCl, 50 mM CaCl_2_). Next, 1 % VinotastePro lysozyme was added to the mycelium and incubated at 25 °C with gentle shaking for 2 h. The resulting protoplasts were harvested by filtration with the sterile filter paper and washed twice with KC buffer. For the transformation of the protoplasts, 10 μg of plasmids or assembled RNPs were added to 1 × 10^7^∼1 × 10^9^ protoplasts. Fifty mM of spermidine was added to the transformation system and incubated on ice for 5 min. Then, 20 % polyethylene glycol (PEG) 3350 was added and mixed at 25 °C for 25 min. The reaction mixture was then spread on NC membrane-covered plates containing regeneration culture medium supplemented with 0.6 M sucrose. After 24 h, the NC membrane was transferred onto plates containing 0.6 M sucrose and an appropriate antibiotic to screen for transformants. A single colony was picked and cultivated, and the genome DNA was extracted using the cetyltrimethylammonium bromide (CTAB) method [39]. Gene editing events were verified by colony PCR using specific primers and then confirmed by DNA sequencing.

### 2.9 RT-qPCR

The transcription of the target genes in the edited strains were detected by RT-qPCR to further confirm the correct edited event. To further confirm the gene transcription pattern in △*bcaba3* and △*bcaba1234* strains, sixteen genes including *BCIN_07g02980*, *BCIN_07g06180*, *BCIN_03g05930*, *BCIN_13g05380*, *bcnde2*, *BCIN_11g06180*, *BCIN_14g01940*, *bcnew1*, *BCIN_14g03610*, *bcPIO4*, *BCIN_05g07680*, *BCIN_09g02130*, *BCIN_03g01280*, *bcddr48*, *BCIN_16g01090* and *bcpio11* were selected and subjected to RT-qPCR.

All the homokaryosis strains were cultured on the PDA solid medium for 6 days and the mycelia were scratched and grounded in liquid nitrogen. All the mycelial RNA was then extracted using TRIzol according to the manufacturer's instructions (Invitrogen, Carlsbad, CA, United States). The quality and concentration of RNA was detected as previously described [21]. cDNA was synthesized by the TaKaRa reverse transcriptase kit (TaKaRa, Dalian, China). The RT-qPCR was conducted using the AceQ qPCR SYBR Green master mix kit (Nanjing Vazyme Biotech Co., Ltd., Nanjing, China). The reactions were conducted in a CFX96 real-time PCR detection system (Bio-Rad, Hercules, CA, United States). Each transcript was analyzed with three independent PCR replicates performed in parallel. The *tubulin* gene (BC1G_05600) was used as an internal control. The fold change (FC) in the mRNA was analyzed by 2^−ΔΔCt^ method. The primers used for RT-qPCR are shown in Table S1.

### 2.10 Quantification of ABA and detection of ABA precursors

The primordial strain *B. cinerea* TB-31 and genetically modified strains (△*bcaba1*, △*bcaba2*, △*bcaba3*, △*bcaba4,* △*bcaba124* or △*bcaba1234*) were separately incubated on PDA medium for 8 days in the dark, followed by acetone extraction. The acetone was then evaporated and the metabolites were solubilized with a mobile phase in a ratio of methanol to water of 7:3. The yield of ABA was detected using high-performance liquid chromatography (HPLC), as previously described [35].

The accumulated ABA precursors such as 1′-deoxy-ABA, and 1′,4′-trans-ABA diol, were detected using UPLC–MS with a Waters ACQUITY UPLC HSS T3 column (2.1 × 100 mm, 1.8 μm). The mobile phase consisted of 0.1 % formic acid in water (solvent A) and acetonitrile (solvent B), with a linear gradient from 20 % to 90 % B over 10 min. Chromatographic separation was achieved at 0.3 mL/min with UV detection at 254 nm and column temperature maintained at 40 °C. Mass spectra were acquired in positive ESI mode, scanning from *m*/*z* 50 to 1000 with a 0.5 s scan interval.

### 2.11 RNA-seq processing and analysis

To comprehensively investigate the differentially expressed genes (DEGs) related to secondary metabolism, the transcriptomes of *B. cinerea* TB-31, △*bcaba3* and △*bcaba1234* were sequenced and compared. After being cultured in a fermentation broth for 6 days at 25 °C, the mycelia of *B. cinerea* TB-31, △*bcaba3*, and △*bcaba1234* were separately collected and filtered. They were then washed three times with phosphate-buffered saline (PBS). Total RNA was isolated using the E.Z.N.A.TM Fungal RNA Miniprep Kit, following the manufacturer's instructions. The quality of the extracted RNA was evaluated using an Agilent 2100 Bioanalyzer (Agilent Technologies, Palo Alto, CA, United States). The cDNA libraries were sequenced on the Illumina sequencing platform by Metware Biotechnology Co., Ltd. (Wuhan, China). The reference genome and its annotation files were downloaded from the NCBI website. Gene expression levels were quantified using feature counts to generate gene alignment statistics. Fragments per kilobase million (FPKM) values were then determined for each gene based on its length. Differential gene expression analysis between the two groups was performed using DESeq2, with P-values adjusted using the Benjamini & Hochberg correction. Genes with a false discovery rate (FDR) parameter below 0.05 and an |log2FC| ≥1 were considered DEGs.

### 2.12 Metabolomics analysis

To assess metabolic flux redistribution, we compared the metabolomes of *B. cinerea* TB-31 with △*bcaba3* and △*bcaba1234* mutants. Strains were cultured in fermentation broth (25 °C, 6 days), harvested by filtration, washed with cold PBS, and lyophilized. Powdered samples (50 mg) were extracted in 1200 μL ice-cold 70 % methanol (with internal standards), centrifuged (12,000 rpm, 3 min), and filtered (0.22 μm). Separation was performed on a Waters ACQUITY UPLC HSS T3 column (40 °C) with a mobile phase of 0.1 % formic acid in water (A) and 0.1 % formic acid in acetonitrile (B), delivered at 0.40 mL/min (4 μL injection volume). The gradient program was 95 % A (initial), ramped to 35 % A over 5 min, shifted to 1 % A at 6 min (held for 1.5 min), and re-equilibrated to 95 % A (2.4 min). Data were acquired in IDA mode (Analyst TF 1.7.1, Sciex). Differential metabolites were identified (VIP >1, |log_2_FC| ≥1).

To detect extracellular terpenoids, 9 mL of TB-31, △*bcaba3*, and △*bcaba1234* fermentation supernatant were collected, vacuum freeze dried and extracted with 500 μL of 70 % methanol (containing an internal standard). Following centrifugation and filtration, sample extracts were analyzed via UPLC-ESI-MS/MS (ExionLC™ AD system; SCIEX) coupled with a tandem mass spectrometer (Metware Biotechnology, Wuhan, China) for targeted terpenoid profiling. Compound identification was performed using in-house MetWare Database (MWDB) with MS/MS spectral matching, while metabolite quantification was carried out via multiple reaction monitoring (MRM) on a triple quadrupole mass spectrometer.

### 2.13 Statistical analysis

Data for RT-qPCR, ABA content and strain growth are presented as mean ± SD from at least three biologically independent replicates. Statistical analyses were performed using GraphPad Prism, with bar charts displaying mean values and error bars representing standard deviations. Detailed analytical methods are provided in the respective figure legends. *P* < 0.05 was considered as significantly different. NS, not significant; ∗, *P* < 0.05; ∗∗, *P* < 0.01; ∗∗∗, *P* < 0.001.

## 3 Results

### 3.1 Establishment of CRISPR/Cas9 gene editing in *B. cinerea* TB-31

To establish CRISPR/Cas9 gene editing in *B. cinerea* TB-31, three different strategies were used. The first strategy involved stable expression of Cas9 in *B. cinerea* TB-31. The nucleic acid sequence encoding *Cas9-EGFP-4NLSs* was cloned and inserted into the expression vector pCBh1 and integrated into the chromosome of *B. cinerea* TB-31 via transformation of the plasmids using ATMT. Fluorescence microscopy revealed that the fusion protein Cas9-EGFP-4NLSs localized to the nucleus of *B. cinerea* TB-31 (Fig. S1), which is a prerequisite for its genome editing. During the initial phase of this study, we aimed to integrate Cas9 into the reported neutral genomic locus of nitrite reductase (*bcniiA*) through homologous recombination. This site has been previously reported to have no effect on fungal growth [11]. We attempted to integrate a 5496 bp Cas9 expression cassette (*Panolic::Cas9*) and the *hygromycin B* resistance expression cassette (*PanTrpC::hph*) into the *bcniiA* locus using 1 kb homologous arms corresponding to the 5′ and 3′ regions of *bcniiA*. However, despite three independent transformation attempts, we were unable to obtain any positive transformants with successful integration at the target locus. This is likely due to the low efficiency of homologous recombination efficiency in filamentous fungi, especially for large DNA fragments (1000 bp + 7380 bp + 1000 bp). Then, we utilized random integration through *Agrobacterium tumefaciens*-mediated transformation (ATMT) to incorporate the *cas9* gene into the genome of *B. cinerea*. This method allowed for the efficient prduction of multiple transgenic strains expressing Cas9 and facilitated the identification of positive transformants. However, the transformation of integrative plasmids containing Cas9-coding sequences consistently resulted in significantly fewer transformants compared to those transformed with empty vector controls (Fig. S2A). Out of 18 randomly selected transformants that were analyzed, 17 showed a significant and variable decrease in ABA content compared to the control strain (TB-31-P) transformed with an empty vector (pCBh1-blank) (Fig. S2B). To further evaluate the effectiveness of these randomly integrated Cas9 strains, we also examined their colonial morphology and genetic stability. The expression of integrated Cas9-4NLSs did not affect colonial morphology (Fig. S3), but it was found to be genetically unstable. PCR verification showed that 16.7 % (4/24) of transformants lost the Cas9 gene after fourth-generation subculturing on antibiotic-containing medium, while all TB-31-P control strains stably retained the empty vector. This instability may be due to the potential cytotoxicity of Cas9, as reported in multiple studies [40,41]. It is possible that the introduction of Cas9 may have pleiotropic effects in *B. cinerea* that extend beyond the observed reduction in ABA biosynthesis. These results suggest that the random integration strategy is not suitable for generating stably modified strains and may pose a potential risk for future industrial applications.

The second strategy involves transient expression of Cas9 by cotransforming plasmids expressing Cas9-4NLSs and sgRNA into the protoplasts of *B. cinerea* TB-31. Plasmids carrying the *A. nidulans* AMA1 sequence, which enables autonomous replication and is rapidly lost under nonselective conditions, are typically used for Cas9/sgRNA expression in filamentous fungi [42]. The Cas9-4NLSs fragment was cloned and inserted into the pAnep8-AMA1 vector and expressed under the constitutive pkiA promoter. The ABA production of twelve randomly selected Cas9 transiently expressed strains was measured. Compared with TB-31, the results revealed no significant effect on ABA yield among these strains (Fig. S4), indicating that the transient expression of Cas9 in the plasmids had a relatively minor effect on the strain. BclaeA, which is a member of the VELVET complex in *B. cinerea* [43], was selected as the target protein to test the editing system due to its distinct phenotypic effects. The △*bclaeA* mutant (representing TB-31 lacking *bclaeA*) exhibited a “marshmallow-like” morphology with white hyphae, providing visual confirmation of successful editing [22]. The sgRNA targeting *bclaeA* was synthesized under the U6 promoter and cloned and inserted into the pJET1.2 vector. Five micrograms of pAnep8-*Cas9*-*4NLSs*-*AMA1* plasmids and 5 μg of pJET1.2-sgRNA-*laeA* plasmids were cotransformed (at a 1:1 ratio) into 1 × 10^8^ protoplasts of *B. cinerea* TB-31, and the transformants were selected through hygromycin B resistance screening. It is worth noting that the pJET1.2-sgRNA-*laeA* plasmid does not contain a fungal selectable marker, and we only used one concentration of hygromycin B for selection. In the end, only 18 transformants were obtained; PCR detection revealed no gene editing, and no significant phenotypic changes were observed in the transformants. These results suggest that the editing efficiency of this strategy was very low.

**Fig. 1:**
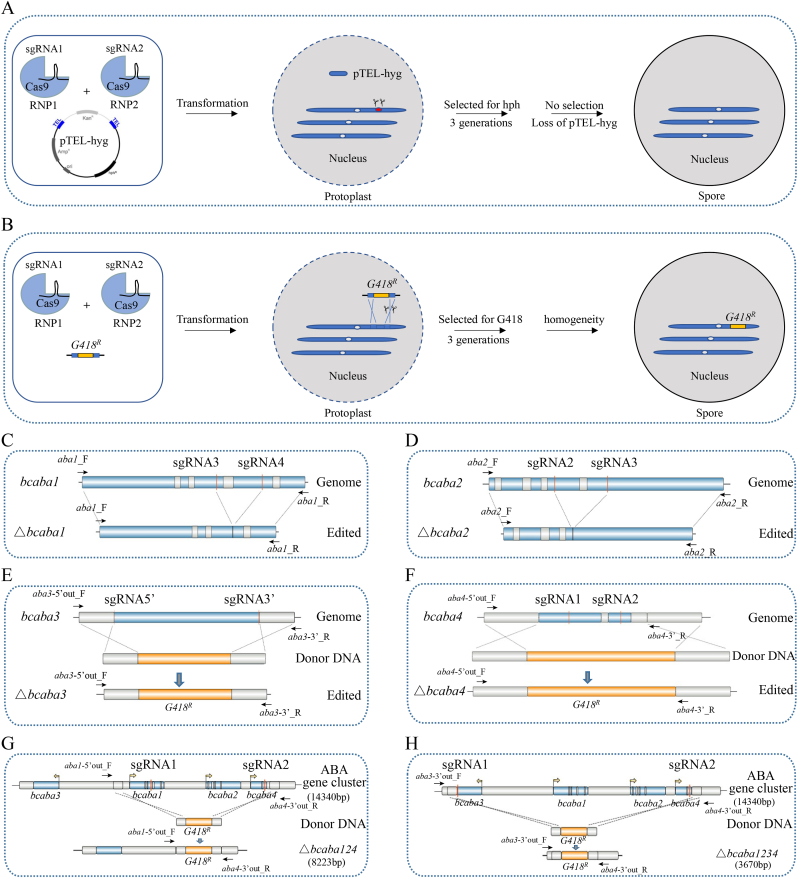
Gene editing strategy using the CRISPR/Cas9 RNP system and the terpenoid synthesis pathway in *B. cinerea* strain TB-31. (A)–(B) Schematic illustration of the workflow of CRISPR/Cas9-mediated gene editing in *B. cinerea* TB-31 using preassembled Cas9-sgRNA RNP complexes. Key steps include the (1) design and synthesis of sgRNAs targeting the gene of interest; (2) *in vitro* assembly of the Cas9 protein and sgRNAs into RNP complexes; (3) delivery of two RNPs and a vector of telomere pTEL-*hyg* (A) or a donor DNA containing a G418 box (B) into *B. cinerea* protoplasts via PEG-mediated transformation; and (4) regeneration and screening of edited strains. (C)∼(F) Genomic diagram of single-gene editing within the ABA BGC. △*bcaba1* (C), △*bcaba2* (D), △*bcaba3* (E), and △*bcaba4* (F); (G)∼(H) Genomic diagram of multigene editing of the ABA BGC. △*bcaba124* (G), △*bcaba1234* (H). Gene structure diagram illustrating the editing events was generated using IBS 2.0 [66], with the gene length scaled down proportionally. The binding locations of the validation primers are indicated by black arrows.

The third strategy involves the transformation of CRISPR-Cas9 RNP complexes into *B. cinerea* TB-31. The workflow for CRISPR/Cas9-mediated gene editing in *B. cinerea* TB-31 using preassembled RNP complexes is shown in Fig. 1A–B. The purified Cas9 and sgRNA target sites are shown in Figs. S5–7. When complexed with sgRNAs to form ribonucleoproteins (RNPs), the high-purity Cas9 protein demonstrates robust cleavage activity *in vitro* (Fig. S8). Ten micrograms of the telomere vector pTEL-*hyg,* which provides transient hygromycin B resistance [33], was cotransformed with the assembled RNPs into 1 × 10^8^ protoplasts of *B. cinerea* TB-31. After screening using selective plates and PCR verification, along with phenotypic characterization, we identified 16 positive transformants with significant large-fragment gene deletions out of the 54 analyzed. These results indicate that the single-gene editing efficiency of this system for *bclaeA* is approximately 30 %. The knockout of the *bclaeA* gene changed the phenotypic characteristics of the colony and decreased the production of ABA (Figs. S9–10), which is consistent with earlier findings [22]. After several transfers on nonselective medium, the telomere vector pTEL-*hyg* was eliminated. Therefore, this system can achieve unmarked editing and is suitable for efficient genetic modification of *B. cinerea* TB-31.

### 3.2 CRISPR/Cas9-mediated single-gene editing of the ABA BGC

To further assess the editing capabilities of the system for other genes, we targeted four genes (*bcaba1*, *bcaba2*, *bcaba3*, and *bcaba4*) from the ABA biosynthesis-related gene cluster using the established CRISPR-Cas9 RNP complex system. Initially, we designed at least two sgRNAs to target the exons in each of the four genes (Fig. S11). The target specificity of the sgRNAs and the cleavage activity of the RNPs were also evaluated *in vitro* (Fig. S12). To increase the editing efficiency, we introduced two pairs of RNPs with high cleavage activity into the protoplasts of *B. cinerea* TB-31 simultaneously to delete the target gene. The editing diagrams are shown in Fig. 1C–F.

Based on the results of *in vitro* validation, two sgRNAs were selected and assembled into RNP complexes for targeted editing of *bcaba1*. The two RNPs were cotransformed with 10 μg pTEL-*hyg* into 1 × 10^8^ *B. cinerea* TB-31 protoplasts, and transformants were selected using hygromycin B. Among the 24 transformants screened, 8 positive candidates were confirmed by diagnostic PCR and sequencing. The editing efficiency for *bcaba1* was approximately 33.3 % in this single study.

For *bcaba2* editing, the two validated high-efficiency sgRNAs were assembled into RNPs and cotransformed with 10 μg of the pTEL-*hyg* vector into 1 × 10^8^ *B. cinerea* TB-31 protoplasts. Eight positive transformants were identified out of the 17 screened through diagnostic PCR and DNA sequencing. The editing efficiency for *bcaba2* was 47.1 % in this study.

Since *bcaba3* lacks introns, sgRNA target sites were designed to flank the 5′ and 3′ ends of its CDS to ensure complete gene deletion. The two RNPs were cotransformed with 10 μg of pTEL-*hyg* into 1 × 10^8^ *B. cinerea* TB-31 protoplasts, yielding 6 positive transformants out of 23. The editing efficiency for *bcaba3* in this study was approximately 26 %. The strategy of *bcaba3* editing was improved by the addition of donor DNA to the transformation system. A fragment of the *AnTrpC* promoter-driven G418 resistance expression cassette (PantrpC::G418), which was obtained from the pCBg418 plasmid [44] and flanked with 300 bp homologous arms of *bcaba3*, was constructed using overlap PCR as the donor DNA. The two RNPs were cotransformed with 5 μg of donor DNA to generate 1 × 10^8^ protoplasts of TB-31, yielding 46 positive transformants (50 in total). The editing efficiency for *bcaba3* increased to more than 90 % with the addition of donor DNA in this study.

Subsequently, the editing of *bcaba4* followed the same strategy as described above. First, the donor DNA of *bcaba4* was constructed with a G418 resistance expression cassette flanked by 500 bp homologous arms of *bcaba4* using overlap PCR. Five micrograms of this donor DNA was introduced into 1 × 10^8^ protoplasts along with two RNPs targeting *bcaba4* for knockout. The resulting editing efficiency of *bcaba4* was 92 %, with 35 out of 38 positive transformants. This high efficiency was achieved with the assistance of the donor DNA.

**Fig. 2:**
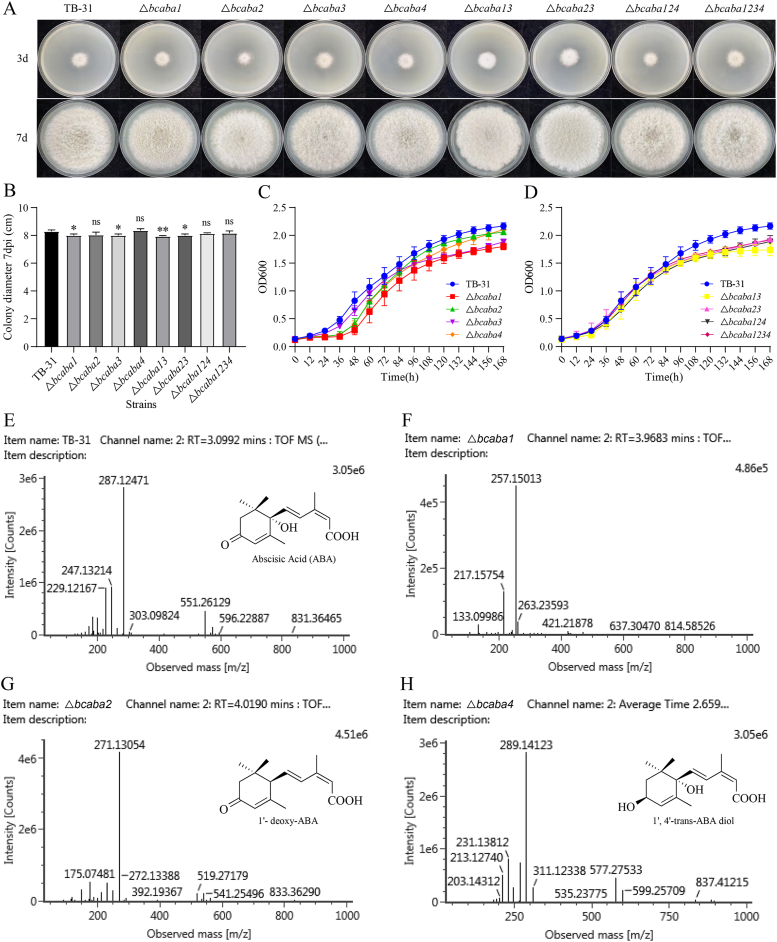
The colony morphologies and diameters, growth curves, and metabolites of modified strains. (A) Colony morphologies of eight modified strains. The *B. cinerea* TB-31 and gene knockout strains were cultured on PDA and photographed after 3 and 7 dpi. (B) Colony diameters of *B. cinerea* TB-31 and the gene knockout strains. *B. cinerea* TB-31 and the modified strains were cultured on PDA plates at 25 °C for 7 days. Data represent medians with interquartile ranges (n = 3). (C)∼(D) Growth curves of *B. cinerea* TB-31 and the gene knockout strains. Optical densities (OD_600_) of the spore suspension of *B. cinerea* TB-31 and the single-gene knockout strains (C) and multigene knockout strains (D) were measured at the indicated time points using a growth analyzer. Data represent the median ± interquartile range (n = 3). (E)∼(H) MS/MS fragmentation spectra of ABA in TB-31 and ABA precursors in △*bcaba1*, △*bcaba2*, and △*bcaba4*. Major fragment ions are labeled with corresponding *m*/*z* values.

The successful deletion of *bcaba1*, *bcaba2*, *bcaba3*, and *bcaba4* in *B. cinerea* TB-31 was confirmed through diagnostic PCR, DNA sequencing, and RT‒qPCR (Figs. S13–14). The edited strains were purified for homokaryosis through third-generation single-spore isolation on resistant PDA solid medium. The colony morphology of *B. cinerea* TB-31 and the four modified strains (△*bcaba1*, △*bcaba2*, △*bcaba3*, and △*bcaba4*, representing *B. cinerea* TB-31 lacking *bcaba1*, *bcaba2*, *bcaba3*, and *bcaba4*, respectively) was observed and photographed at 3 and 7 days post-inoculation (dpi) (Fig. 2A). The four modified strains exhibited light gray pigmentation and aerial hyphal morphology comparable to that of TB-31, with no statistically significant difference (P > 0.05) or only marginal variation in colony diameter (Fig. 2B). The growth curves of these obtained strains in fluid medium were measured by analyzing the optical density at 600 nm. The growth curves of these mutants closely overlapped with those of the wild-type strain (Fig. 2C), indicating that the single knockout of the ABA biosynthesis gene did not significantly affect the growth of *B. cinerea* TB-31. These results are consistent with the observations from the colony diameter on the PDA solid plate culture experiments.

The major metabolites of the modified strains *bcaba1*, △*bcaba2*, △*bcaba3,* and △*bcaba4* were analyzed using UPLC-MS. In contrast to the wild-type *B. cinerea* strain TB-31, the predominant metabolites detected in the △*bcaba2* and △*bcaba4* mutants were identified as 1′-deoxy-ABA and 1′,4′-trans-ABA diol, respectively. An unknown compound was detected at a retention time of 3.96 min in △*bcaba1* strain by mass spectrometry, displaying a peak at [*m*/*z*] 257.15031. This compound has been tentatively characterized as α-(γ)-ionylidene acetic acid in previous literature [25]. Siewers et al. suggested that 1′-deoxy-ABA in △*bcaba2* strain may represent a side product of the native pathway, resulting from nonspecific enzymatic conversion of 4′–OH–α-ionylideneacetic acid when C-1′ hydroxylation is impaired [45] (Fig. 2E–H) (Fig. S15A–D). Neither ABA nor its structural analogs were detected in the △*bcaba3* strain with the same method, indicating a complete blockage of ABA biosynthesis (data not shown). These results further confirmed the complete loss of function of the target gene.

### 3.3 CRISPR/Cas9-mediated multigene editing in *B. cinerea* TB-31

To evaluate the system's capacity for dual-gene knockout, we initially used a step-by-step experimental approach. We started with the previously generated △*bcaba1* and △*bcaba2* strains and deleted the *bcaba3* gene to obtain the △*bcaba13* and △*bcaba23* strains*.* The donor DNA, containing a G418 expression cassette flanked by *bcaba3* homologous arms, was constructed and cotransformed with RNPs targeting the 5′- and 3′- ends of the *bcaba3* CDS. Five micrograms of donor DNA and two assembled RNPs targeting *bcaba3* were cotransformed into 1 × 10^8^ protoplasts of the △*bcaba1* or △*bcaba2* strain. The △*bcaba13* and △*bcaba23* strains were successfully obtained through selection using hygromycin and G418 resistance markers, with an efficiency of approximately 90 %. These results demonstrate that sequential gene knockout can be achieved using this stepwise approach.

To assess the ability of the system to delete large fragments, we designed sgRNAs to target three adjacent genes (*bcaba1*, *bcaba2*, and *bcaba4*) to achieve their simultaneous knockout in a single-step approach (Fig. 1G). First, we selected two sgRNAs targeting *bcaba1* and *bcaba4* and assembled them into RNPs. Afterward, we amplified two fragments—one spanning from −864 bp to −364 bp upstream of the initiation codon of *bcaba1* and the other spanning from +148 bp to +647 bp downstream of the stop codon of *bcaba4*—as 5′- and 3′-homologous arms, respectively. Next, we obtained the donor DNA, which contains an *AnTrpC* promoter-driven G418 resistance expression cassette flanked by a 5′ homologous arm and 3′ homologous arms, using overlap PCR. We then cotransformed 1 × 10^8^ TB-31 protoplasts with 5 μg of the donor DNA and the two efficient RNPs separately targeting *bcaba1* and *bcaba4.* Through G418 resistance screening, we obtained 33 transformants in a single transformation, all of which were confirmed to carry the correct deletion. The △*bcaba124* strain was further analyzed through PCR-based genomic diagnosis and sequencing (Fig. S13E). With the help of the donor DNA, we achieved nearly 100 % efficiency in deleting a long segment (5897 bp) in the △*bcaba124* strain.

We utilized the same strategy to remove the entire 13.5 kb gene cluster region containing the four genes *bcaba1*, *bcaba2*, *bcaba3*, and *bcaba4*. The donor DNA, which consisted of a G418 expression cassette flanked by two 500 bp homologous arms, was obtained. The 5′ homologous arm spanned from +243 bp to +742 bp downstream of the stop codon of *bcaba3*, whereas the 3′ homologous arm spanned from +148 bp to +647 bp downstream of the stop codon of *bcaba4* (Fig. 1H). The donor DNA, along with RNPs assembled with two verified sgRNAs targeting *bcaba3* and *bcaba4*, were cotransformed into 1 × 10^8^ TB-31 protoplasts. Thirty transformants were selected and analyzed using genomic diagnostic PCR, with twenty-eight showing the desired deletion (Fig. S13F). The efficiency of the gene editing system for long-segment deletion was greater than 90 %, resulting in the △*bcaba1234* strain. Both the colony morphology and growth characteristics of the multigene knockout strain were comparable to those of TB-31, with either no significant difference (P > 0.05) or only minor variations observed (Fig. 2A, B, D). The relative transcription levels of the target genes in the constructed modified strains were determined via RT‒qPCR, the results of which were consistent with the earlier genomic PCR validation results, confirming the successful construction of the modified strains (Fig. S14E‒F).

### 3.4 Transcriptome analysis of the △*bcaba3* and △*bcaba1234* strains

**Fig. 3:**
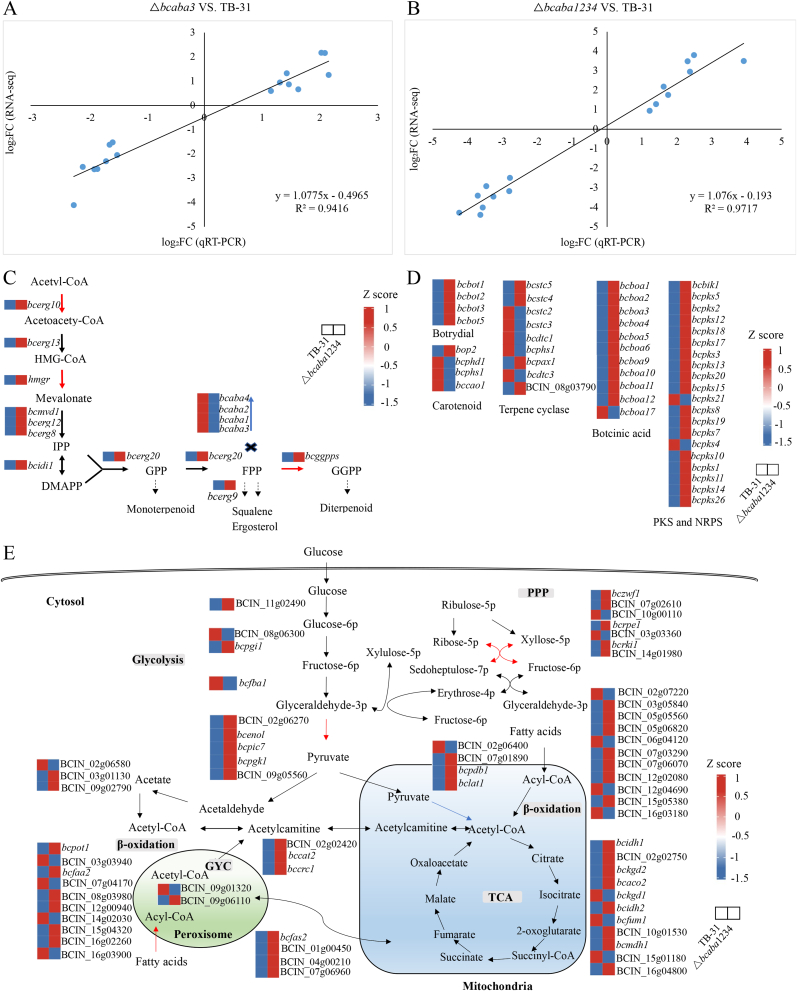
Transcriptomic analysis of *B. cinerea* strains TB-31, △*bcaba3*, and △*bcaba1234*. (A)∼(B) Regression analysis between transcriptomic data (RNA-seq) and RT‒qPCR validation. △*bcaba3* vs. TB-31: R^2^ = 0.94, *P* < 0.001 (A); △*bcaba1234* vs. TB-31: R^2^ = 0.97, *P* < 0.001 (B). (C) Heatmap of the relative transcriptional levels of the genes in the MVA pathway in △*bcaba1234* compared with those in TB-31. Gene transcription levels were normalized using Z score transformation, calculated as (X-mean)/SD, to represent the number of standard deviations above or below the mean transcription level. Upregulated genes are highlighted in red, while downregulated genes are shown in blue. The data are derived from three independent biological replicates. (D) Heatmap of the relative transcriptional levels of DEGs involved in other secondary metabolism pathways in △*bcaba1234* compared with those in TB-31. (E) Heatmap of the relative transcriptional levels of genes involved in intracellular primary metabolic pathways in the △*bcaba1234* strain compared with those in the TB-31 strain. Metabolic pathways are marked with gray boxes. PPP: pentose phosphate pathway; TCA: tricarboxylic acid cycle; GYC: glyoxylate cycle.

The generated mutants, strains △*bcaba3* and △*bcaba1234*, exhibited complete abolition of ABA and its structural analog precursors. To clarify the effects of the absence of the *bcaba3* gene and ABA gene clusters on the transcription levels of terpene biosynthesis-related genes, we analyzed the transcriptomes of the two strains. We selected the early stage of ABA synthesis for RNA-seq analysis. In comparison to *B. cinerea* TB-31, △*bcaba3* had a total of 688 DEGs, with 335 genes being upregulated and 353 genes being downregulated. In contrast, △*bcaba1234* showed 1501 DEGs, with 736 genes being upregulated and 765 genes being downregulated (Fig. S16). Regression analysis of the RNA-seq and RT‒qPCR data revealed a strong correlation (△*bcaba3* vs. TB-31: R^2^ = 0.94; △*bcaba1234* vs. TB-31: R^2^ = 0.97, P < 0.001 for both), which demonstrated excellent agreement between the two methods (Fig. 3A–B) and validated the reliability of the transcriptomic data.

Since ABA synthesis in *B. cinerea* is derived from the MVA pathway, we first analyzed the changes in the expression levels of genes in the MVA pathway. Acetyl-CoA C-acetyltransferase (Bcerg10, BCIN_05g07430), HMG-CoA reductase (Hmgr, BCIN_05g03150), and geranylgeranyl pyrophosphate synthetase (GGPPS, BCIN_05g05660 and BCIN_05g05670) were markedly upregulated among the DEGs in the △*bcaba1234* strain compared with those in the TB-31 strain (Fig. 3C), while the transcription levels of these genes showed no significant changes in the △*bcaba3* strain (Tables S2–3). Thus, we focused on the △*bcaba1234* strain and further analyzed the DEGs involved in other related secondary metabolism pathways compared with those in TB-31 (Fig. 3D). Within the sesquiterpenoid botrydial BGC, the expression of *bcbot1* and *bcbot2* was upregulated, whereas the expression of the remaining genes in this cluster did not significantly change. In contrast, the expression of the carotenoid biosynthesis-related genes *bcphs1* (BCIN_01g04560) and *bccao1* (BCIN_01g04570), encoding phytoene dehydrogenase and carotenoid cleavage dioxygenase 1, respectively, decreased. Among the other terpene synthases, the transcript level of *bcstc2* (BCIN_08g02350) was downregulated, while that of *bcpax1* (BCIN_05g05670) was upregulated. The transcript levels of 10 genes involved in botcinic acid biosynthesis were upregulated, whereas that of *bcboa17* was downregulated. Botcinic acid belongs to the polyketide class of secondary metabolites, and its biosynthesis also originates from acetyl-CoA.

The transcriptional profiles of primary metabolic pathway genes closely associated with terpene synthesis-related pathways in the △*bcaba1234* strain, including carbohydrate transport and utilization, acetyl-CoA synthesis and transport-related pathways, the pentose phosphate pathway, and fatty acid biosynthesis, were subsequently analyzed (Table S4). The central metabolite acetyl-CoA is the link between the primary metabolic pathway and the secondary metabolic pathway and is also the precursor of terpene synthesis in *B. cinerea*. Pyruvate is an important node in the synthesis of acetyl-CoA. Both glycolysis and the pentose phosphate pathway (PPP) convert glucose into intracellular pyruvate. Therefore, the relative transcriptional abundance of the genes in these pathways was analyzed. First, DEGs in these pathways were selected and analyzed. The levels of transcription for phosphoribosyl pyrophosphate synthetase (PRPP synthetase, BCIN_06g00790), which catalyzes the conversion of ribose-5-phosphate (R5P) into 5-phospho-α-d-ribose-1-diphosphate (PRPP) in the PPP pathway, were significantly greater in the △*bcaba1234* strain than in the TB-31 strain. Concurrently, we detected upregulated expression of nicotinate-nucleotide adenylyltransferase (BCIN_13g01400) and NAD kinase (BCIN_06g03210), key enzymes in the nicotinamide pathway that maintain intracellular NAD^+^ pools essential for redox reactions, in the △*bcaba1234* strain. On the other hand, the transcription level of pyruvate carboxylase Bchfa1 (BCIN_02g01460) in the tricarboxylic acid (TCA) cycle, which consumes acetyl-CoA, was downregulated in the △*bcaba1234* strain compared with that in the TB-31 strain. The transcription patterns of genes involved in these related metabolic pathways are shown through a heatmap of z scores in the △*bcaba1234* strain compared with the TB-31 strain based on transcriptomic data (Fig. 3E). Integrated analysis of gene expression heatmaps and differential expression of key genes in the △*bcaba1234* strain revealed metabolic flux redistribution involving the following adaptive adjustments: increased upstream supply, with increased flux through the pentose phosphate pathway (PPP) to provide more reducing power and precursor metabolic substrates; safeguarded redox balance via activation of the nicotinamide pathway to maintain NAD^+^ pool homeostasis to support high metabolic activity; and optimized downstream shunting, where reduced consumption of acetyl-CoA by the tricarboxylic acid (TCA) cycle directs it preferentially toward the mevalonate (MVA) pathway for terpenoid synthesis. Ultimately, this redistribution enables cells to focus metabolic resources on terpenoid precursor production following blocked ABA synthesis, reflecting the adaptive remodeling of the fungal metabolic network in response to the loss of a key synthetic pathway.

### 3.5 Metabolomic analysis of the △*bcaba3* and △*bcaba1234* strains

**Fig. 4:**
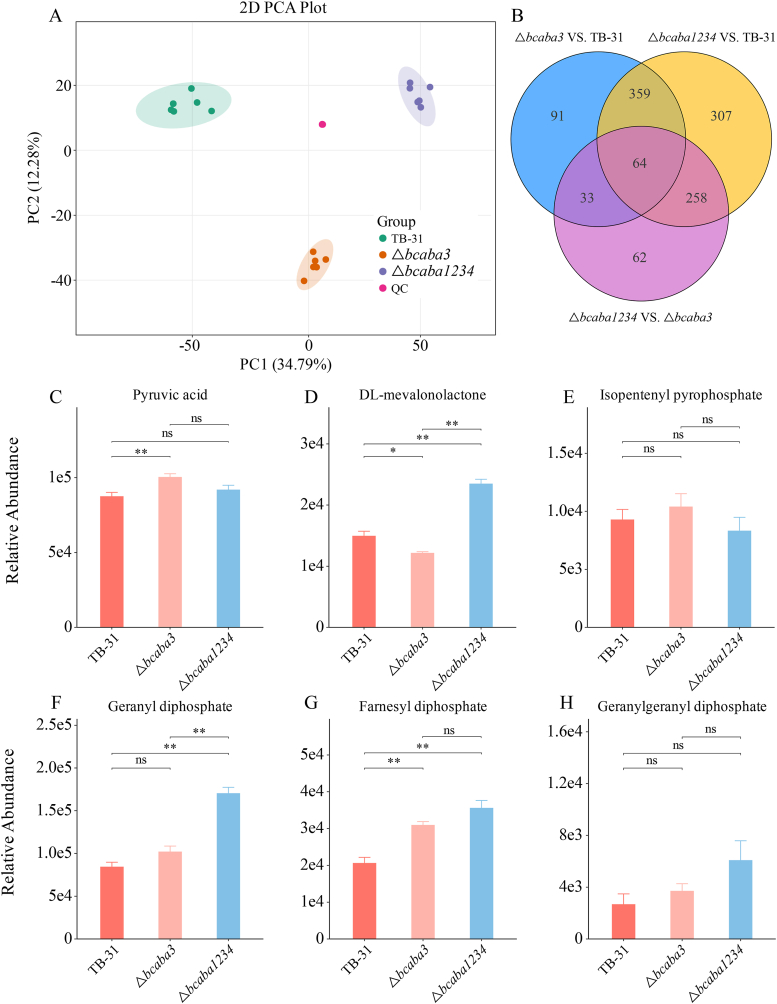
Nontargeted metabolomics analysis of the △*bcaba3* and △*bcaba1234* strains compared with TB-31. (A) PCA of metabolomic profiles, showing distinct clustering of the control strain TB-31 and the mutant strains △*bcaba3* and △*bcaba1234*. Each point represents a biological replicate, and the percentage of variance explained by each principal component (PC1 and PC2) is indicated on the axes. (B) Venn diagram illustrating the overlap of differentially abundant metabolites (DAMs) among the three strains. DAMs were identified using thresholds of |log2 FC| > 1 and adjusted p value (FDR) < 0.05. (C)∼(H) Relative content of key terpenoid biosynthesis precursors in △*bcaba3* and △*bcaba1234* compared with that in TB-31. The relative contents of key terpenoid biosynthesis precursors, including pyruvic acid (C), mevalonolactone (D), IPP (E), GPP (F), FPP (G) and GGPP (H), were quantified and compared among the strains. The data are presented as the means ± SEMs of three independent biological replicates. Statistical significance was determined using one-way ANOVA. ∗, *P* < 0.05; ∗∗, *P* < 0.01; ∗∗∗, *P* < 0.001.

To further verify how the absence of ABA biosynthesis affects changes in primary and secondary metabolic fluxes within cells, we analyzed the untargeted metabolomic profiles of the △*bcaba3* and △*bcaba1234* strains in comparison with that of TB-31. Principal component analysis (PCA) revealed tight clustering of the six biological replicates, indicating high reproducibility, while distinct separation between groups reflected significant metabolic differences (Fig. 4A). A total of 4353 metabolites were identified in both positive and negative ionization modes. Compared with TB-31, the △*bcaba3* strain presented 534 significantly differentially expressed metabolites, with 214 upregulated and 320 downregulated metabolites. A more pronounced difference in metabolic alterations was observed in △*bcaba1234*, with 977 significantly differentially expressed metabolites compared with those in TB-31. Of these, 486 were upregulated, and 491 were downregulated. When △*bcaba1234* and △*bcaba3* were directly compared, 396 significantly differentially expressed metabolites were identified, including 166 upregulated and 230 downregulated metabolites (Fig. S17A–C). A Venn diagram illustrating differentially abundant metabolites among the experimental groups is shown in Fig. 4B.

Metabolic profiling revealed significant alterations in key pathways. Specifically, the levels of glycolysis intermediates (α-d-glucose-6P and d-fructose-6P) were markedly greater than those in TB-31. Additionally, terpenoid backbone biosynthesis showed an accumulation of mevalonate-5P (MVA-5P) and farnesyl pyrophosphate (FPP), while diterpenoid biosynthesis exhibited a pronounced increase in stevioside content. Furthermore, the biosynthesis of ubiquinone and terpenoid-quinone increased the levels of phytyl-diphosphate, polyprenyl-PP, and chorismate. Quantitative analysis revealed that key metabolic intermediates of the MVA pathway, including GPP, FPP, and GGPP, significantly accumulated in both mutant strains compared with *B. cinerea* TB-31. Notably, the △*bcaba1234* strain exhibited the most pronounced accumulation of these isoprenoid precursors among the studied strains (Fig. 4C–H). These findings suggest that, compared with TB-31, the △*bcaba1234* strain has upregulated glycolysis, potentially due to increased energy demand or altered carbon flux, as well as higher mevalonate pathway activity. Collectively, these shifts resulted in a systemic reorientation of metabolic flux toward terpenoid production.

Comparative enrichment of KEGG pathways was detected by both transcriptomic and metabolomic profiling in △*bcaba1234* compared with TB-31. The bars represent the -log10(p value) from hypergeometric testing (Fig. S18). The enriched DEGs and differentially abundant metabolites showed high overlap consistency in glycerophospholipid metabolism, terpenoid backbone biosynthesis, diterpenoid biosynthesis and ubiquinone and other terpenoid-quinone biosynthesis; this may be evident from the upregulated relative transcription level of key MVA genes and the accumulation of isoprenoid precursors in the △*bcaba1234* strain.

**Fig. 5:**
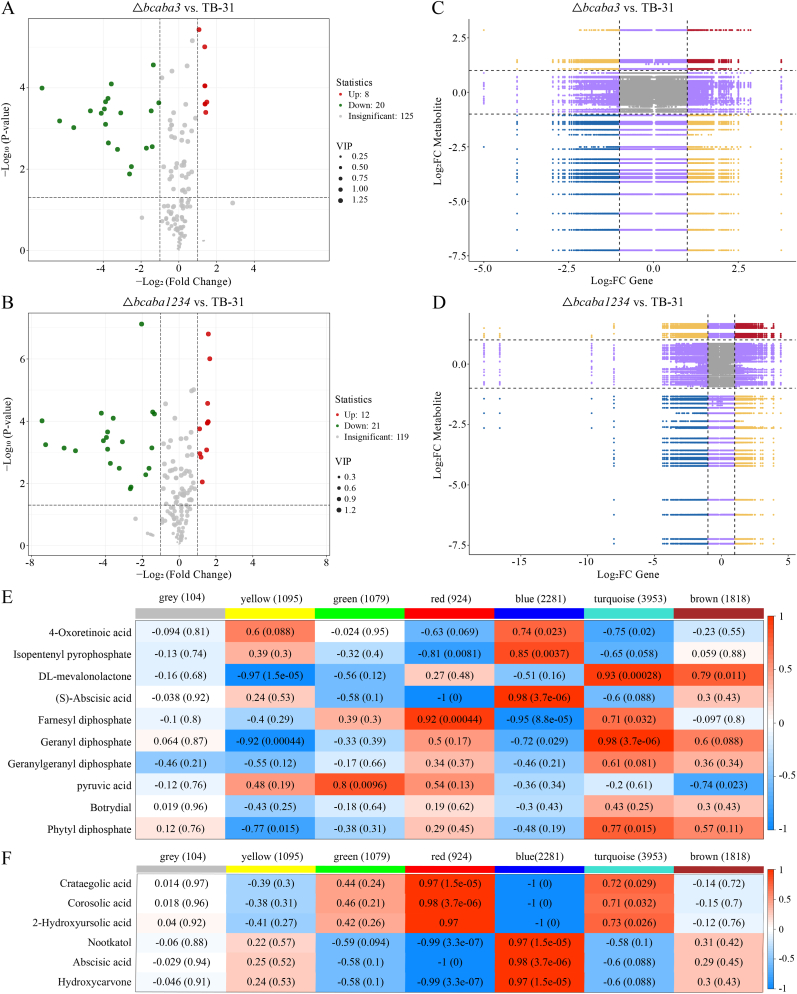
Widely targeted metabolomics of extracellular terpenoid biosynthesis and integrated transcriptomic-metabolomic analyses. (A)∼(B) Volcano plot of differentially abundant metabolites in △*bcaba3* vs. TB-31 (A) and △*bcaba1234* vs. TB-31 (B). Differentially abundant metabolites were screened based on VIP, FC, and statistical significance (*P* value). Red and green points denote significantly upregulated and downregulated metabolites (VIP >1, FC ≥ 2 and FC ≤ 0.5, *P* < 0.05), respectively; gray points indicate nonsignificant metabolites. The x-axis represents the log_2_FC (fold change in relative abundance). (C)∼(D) Nine-quadrant correlation analysis of transcriptomic and terpenoid metabolomic data. The plot compares differential expression patterns of genes and metabolites between the △*bcaba3* vs. TB-31 (C) and △*bcaba1234* vs. TB-31 comparisons (D) Quadrants 3 and 7 represent concordant expression patterns, while quadrants 1 and 9 represent antagonistic expression patterns. (E)∼(F) WGCNA of key differential metabolites and associated gene modules. (E) Integration of the transcriptome with non-targeted metabolomics data. (F) Integration of the transcriptome with targeted terpenoid metabolomics data.

To further investigate whether metabolic flux was redirected to the biosynthesis of other terpenoids in these ABA-blocked strains post-fermentation, a comprehensive widely targeted metabolomics analysis of extracellular terpenoids in the fermentation broth was performed. A total of 153 terpenoid metabolites were identified using the UPLC‒MS/MS ESI platform in combination with the custom-built metabolite database MWDB. The terpenoids detected included 51 monoterpenes, 39 sesquiterpenes, 18 diterpenes, and 45 triterpenes. Comparative analysis revealed distinct metabolic profiles across the groups: compared with the TB-31 group, the △*bcaba3* group presented 28 significantly altered metabolites, comprising 20 downregulated and 8 upregulated metabolites. Similarly, compared with the TB-31 group, the △*bcaba1234* group displayed 33 significantly different metabolites, with 21 downregulated and 12 upregulated (Fig. 5A–B).

Compared with those in strain TB-31, the relative contents of fifteen sesquiterpenes, including nootkatol, michelenolide, and spathulenol, and three triterpenes, namely, ganoderic acid DM, ganoderic acid S and zizyberanal acid were decreased in △*bcaba1234*. In contrast, the relative contents of one monoterpenoid, 8-hydroxy-1,7-epoxy-2-menthene, and four sesquiterpenoids, 9-hydroxy-3,10(14), 11-germacratrien-15-al, 11-hydroxy-3-oxo-4(5), 6(7)-diene-eudesman-12-ol, 9,10-dihydroxy-4-megastigmen-3-one, and 4,9-dioxo-5,7-megastigmadien-11-oic acid, increased. Additionally, the levels of seven triterpenes, including crataegolic acid, 2-hydroxyursolic acid, and corosolic acid, also increased. By using the Z score to standardize the relative content of differentially abundant metabolites across different samples, the distribution of each differentially abundant metabolite among various groups can be easily visualized in a highly intuitive manner (Fig. S19). These findings indicate that in the ABA BGC knockout strain, the metabolic flux within the cells is redistributed, leading to an altered abundance of terpenoid metabolites secreted extracellularly.

Integrated transcriptomic and metabolomic data were analyzed using nine-quadrant correlation plot, revealing the differential expression patterns of genes and metabolites in the △*bcaba3* and △*bcaba1234* mutants relative to the TB-31 strain (Fig. 5C–D). Our non-targeted metabolomic profiling of intracellular metabolites identified multiple compounds that were differentially associated with terpenoid biosynthesis. We then performed Weighted Gene Co-expression Network Analysis (WGCNA) using the most significantly altered metabolites and their correlated genes (Fig. 5E). This analysis grouped the correlated genes into distinct modules. For example, in the association analysis with (S)-abscisic acid, two gene modules, designated blue and red, exhibited strong correlations (|r| ≈ 1), comprising 2281 and 924 genes, respectively. We also used WGCNA in the integrative analysis of the transcriptomic and extracellular terpenoid metabolomic data for the selected six significantly different terpene metabolites: Crataegolic acid; Corosolic acid; 2-Hydroxyursolic acid; Nootkatol; Abscisic acid and Hydroxycarvone (Fig. 5F). We then subjected these correlated genes to KEGG pathway annotation, and constructed metabolite-gene correlation networks. Based on the terpenoid metabolomics analysis of the △*bcaba1234* vs. TB-31 strains, we identified correlated terpenoid products from the metabolite correlation heatmap (Fig. S20). In the integrated transcriptomic and metabolomic analysis of the △*bcaba1234* vs. TB-31 comparison, we constructed correlation networks for two key differentially abundant metabolites: ABA, which was significantly reduced, and crataegolic acid, which was markedly increased, along with their significantly correlated genes (Figs. S21–S22). This set of filtered genes provides a basis for further analysis of endogenous genes involved in the biosynthesis of these terpenoids. Our analyses clearly revealed a set of genes significantly associated with endogenous terpenoids and their biosynthetic precursors. These genes provide a strategic starting point for further investigation into the molecular mechanisms underlying terpenoid metabolism.

This metabolic profile further indicates that the △*bcaba1234* strain, with accumulated terpene biosynthesis precursors and an active metabolic environment for the synthesis of terpenoids, could serve as a promising platform for the direct enhancement of competitive terpene synthesis.

### 3.6 CRISPR/Cas9-based targeted integration system in *B. cinerea* TB-31

**Fig. 6:**
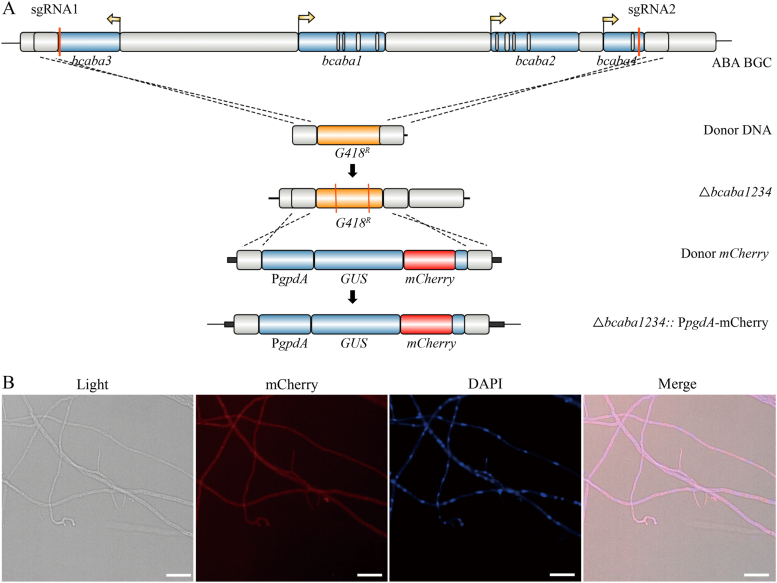
CRISPR/Cas9-based targeted integration system. (A) Genomic diagram of mCherry-targeted integration. (B) Fluorescence microscopy images showing the efficient expression of mCherry-targeted integration in the △*bcaba1234* strain with 5-day-old germlings on glass slides and nuclei stained with DAPI (blue). Scale bar: 20 μm.

To explore whether this system can be applied for targeted gene integration and controlled expression of exogenous genes, we developed a CRISPR/Cas9-based unmarked targeted integration system that utilizes pTEL-hyg and donor DNA. In our preliminary validation experiments, we used the red fluorescence protein mCherry as the reporter gene for integration at the *G418* locus in the strain △*bcaba1234*. Two sgRNAs were designed to target the *G418* CDS in the △*bcaba1234* strain (Fig. 6A). The donor DNA, which consisted of the *mCherry* box flanked by 5′- and 3′- homologous arms located above and below the *G418* locus, was constructed using overlap PCR. After the cotransformation of RNPs, 5 μg of donor DNA, and 5 μg of pTEL-*hyg*, we were able to select correct targeted integration transformants on a hygromycin plate, with a positive rate of approximately 40 % (25/62). With the disruption of the G418 resistance gene, the obtained strain was passaged three to five times on medium without antibiotics to eliminate the pTEL-hyg plasmid, ultimately yielding an antibiotic marker-free strain expressing the exogenous protein mCherry. The number of passages required for the loss of the pTEL transient selection plasmid varies among different genes. Generally, after 3 to 5 passages in an antibiotic-free medium, the plasmid is lost in the edited strains [33]. The presence of red fluorescence indicated the successful insertion and expression of mCherry (Fig. 6B).

These results demonstrate that the CRISPR/Cas9 system established in this study enables precise targeted integration, making it a versatile tool for endogenous and heterologous gene expression in *B. cinerea*.

## 4 Discussion

*B. cinerea* has been widely used in industry for the fermentative production of ABA, due to its inherent advantages in scaling up terpenoid biosynthesis. The aim of this study was to establish and optimize the CRISPR/Cas9 gene editing system in the ABA-producing strain *B. cinerea* TB-31 to expand its metabolic engineering toolkit for iterative strain improvement and broaden its capacity for the biosynthesis of other high-value terpenoids beyond ABA. Through our research, we successfully established and improved the RNP-based CRISPR/Cas9 system in *B. cinerea* strain TB-31 and generated several modified strains in which single or multiple genes of the ABA BGC were knocked out. Our results showed that using two RNPs to target a single gene or gene cluster resulted in increased editing efficiency, especially when the RNPs were cotransformed with the donor DNA flanked by shorter homologous arms. Our deletion of the entire ABA BGC led to upregulation of the MVA pathway and increased levels of terpenoid biosynthetic precursors. Furthermore, this approach can be used for site-specific genome integration in this strain, which is crucial for the stable expression of key genes and metabolic pathway engineering.

The CRISPR/Cas9 genome editing system has been applied in filamentous fungi for genetic engineering and metabolic engineering modifications. However, due to its multinucleate nature, extremely low homologous recombination efficiency, and limited availability of selectable markers, the CRISPR/Cas9 genome editing system was not established in the important necrotrophic plant pathogen *B. cinerea* B05.10 until 2020 [33]. To date, no successful application has been reported in industrially relevant *B. cinerea* strains, which have more complex genomic architectures. The delivery of CRISPR/Cas9 system expression plasmids or RNP complexes into filamentous fungal cells is essential for effective genome editing. In our initial research, we integrated the Cas9-4NLSs into the genome for continuous gene expression, but this process may exhibit potential cytotoxicity to the host strain. Many studies have used plasmids that contain the autonomously replicating sequence AMA1 for transient expression of Cas9 in filamentous fungi [46]. Co-transformation is a well-established procedure for filamentous fungi, as demonstrated in the model species *Aspergillus nidulans* [47]. Our study utilized this technique by thoroughly mixing two different plasmids in equal proportions during protoplast transformation of *B. cinerea*. We were able to select transformants using only one antibiotic resistance marker, hygromycin B, by adopting a co-transformation strategy with 1:1 ratio of pAnep8-*Cas9-4NLSs-AMA1* and pJET1.2-sgRNA-*laeA* plasmids. This approach has been successfully utilized in the CRISPR/Cas9-mediated genome editing system of the thermophilic fungus *Myceliophthora* species, demonstrating its effectiveness [48]. During the construction of our transient Cas9 expression system, we encountered an issue with the size of the plasmid when cloning Cas9 into a plasmid containing the AMA1 element. The resulting plasmid was 15,724 bp, which could potentially hinder its transformation efficiency into filamentous fungal protoplasts. At the same time, we also created a plasmid that contain Cas9, the sgRNA cassette, and the AMA1 element, with a total size of 16,569 bp. The sgRNA cassette included the U6 promoter, a 20 bp protospacer sequence targeting *bclaeA*, and the sgRNA scaffold. However, when we attempted to transform *B. cinerea* protoplasts with this plasmid, we obtained very few transformants (<10), none of which exhibited phenotypic changes. This low transformation efficiency could be attributed to both the large size of the plasmid constructs and the inherent difficulty of transforming multinucleated *B. cinerea*. The size of the plasmid was demonstrated to be inversely correlated with transformation efficiency [49]. One study revealed that a truncated AMA1 variant (reduced from 5.3 kb to 2.8 kb) retained its biological function and exhibited high transformation efficiency in *A. niger* [50], indicating its potential for improving delivery efficiency. RNPs, which are formed by purified Cas9 and extracellularly transcribed sgRNA, provide an alternative CRISPR/Cas9 delivery method and have become more favorable for filamentous fungal studies [[51], [52], [53]]. In this study, the RNP-mediated CRISPR/Cas9 system enabled precise single-gene editing in the ABA-producing strain *B. cinerea* TB-31 with 90 % efficiency when donor DNA containing 300–500 bp homology arms was used. This efficiency matches that achieved in the well-characterized model filamentous fungus *A. oryzae* [54]. Furthermore, large fragments (entire BGCs) can be deleted in a single transformation, making the study of the BGCs in *B. cinerea* more effortless.

Through comprehensive phenotypic characterization of strain TB-31 and its mutants, including growth assays, transcriptomics, and metabolomics profiling, we systematically evaluated the impact of ABA gene cluster knockout on (i) the expression of MVA pathway genes, (ii) intracellular metabolic flux redistribution, (iii) product spectrum and titers, and (iv) intermutation variability. The investigation of transcriptome and metabolomic of the generated mutants, strains △*bcaba3* and △*bcaba1234*, revealed some novel information. These insights primarily fall into the following two aspects: (1) By knocking out the entire ABA biosynthetic gene cluster, we were able to successfully redirect metabolic flux and alter the allocation of terpenoid precursors. While some metabolic flux was partially diverted into minor endogenous terpenoids in the △*bcaba1234* strain, there was a general accumulation of universal terpenoid precursors. Our analysis, which integrates transcriptome and metabolome data, has revealed a set of genes that are significantly related to endogenous terpenoids and their biosynthetic precursors. This provides a foundation for future metabolic engineering strategies, such as inhibiting competitive metabolic pathways. (2) The △*bcaba1234* strain exhibited a greater number of differentially expressed genes and differential metabolites compared to △*bcaba3*, along with elevated levels of terpenoid biosynthesis precursors. This suggests that although both strains lost the ability to produce ABA, the knockout of the entire ABA biosynthetic gene cluster in △*bcaba1234* induced more extensive metabolic reprogramming and cellular perturbation. This comparative analysis highlights the regulatory significance of ABA synthesis genes in terpene synthesis, which is crucial for understanding the regulatory mechanisms of ABA and other terpene synthesis in *B. cinerea.*

The upregulation of key genes in the MVA pathway and increased accumulation of terpenoid precursor metabolites observed in the △*bcaba1234* strain may be attributed to the following: blocked ABA biosynthesis not only weakens the feedback inhibition of ABA on the synthesis of MVA pathway precursors (e.g., GPP and FPP) but also redirects more precursors originally destined for ABA toward other terpenoid biosynthesis branches, ultimately resulting in enhanced metabolic flux diversion to other terpenoids. Therefore, we hypothesize that the △*bcaba1234* strain is well suited as a chassis for terpenoid biosynthesis and holds potential for the production of other high-value terpenoids in addition to ABA. The complete elimination of the BGC ensures the removal of any possible genetic crosstalk, thereby providing a more robust experimental system for pathway analysis. The removal of endogenous BGCs liberates key substrates, including acetyl-CoA, for utilization in heterologous pathways while simultaneously reducing metabolic complexity, thereby enhancing the detection and isolation of novel compounds. Similarly, Chiang et al. extended their strategy by deleting the entire >50 kb sterigmatocystin (ST) BGC in *A. nidulans* to construct strain LO4389, as although core gene deletion alone abolishes ST production, residual BGC genes might still interfere with metabolic pathways [55].

Currently, filamentous fungi are receiving increasing attention as chassis organisms for terpenoid production, particularly for complex fungal-derived terpenoids. Compared with traditional model chassis such as *Escherichia coli* and *Saccharomyces cerevisiae*, filamentous fungi exhibit distinct advantages for this purpose. For instance, microorganisms such as *E. coli* and yeast species (*S. cerevisiae* and *Yarrowia lipolytica*) have been employed in the synthesis of many high-value terpenoids, including farnesene, amorphadiene, lycopene and β-carotene [1]. However, *E. coli* lacks eukaryotic posttranslational modification machinery, which makes it difficult to synthesize complex oxidative derivatives, while yeast strains often exhibit inefficient splicing of filamentous fungal mRNA [56]. Additionally, many secondary metabolites from filamentous fungi are cytotoxic to *S. cerevisiae* [7]. In contrast, higher fungi serve as a superior platform for the biosynthesis of these complex terpenoids, as they possess a fully developed endomembrane system and redox partner proteins, enabling efficient expression of fungal-derived terpene synthases and cytochrome P450 oxidases [57]. For example, Yuan et al. developed an automated high-throughput (auto-HTP) biofoundry workflow in *Aspergillus oryzae* by simultaneously refactoring 39 BGCs into 208 engineered strains and successfully produced 185 distinct terpenoids, demonstrating the immense potential of filamentous fungi in synthesizing complex fungal-derived terpenes [58]. *Trichoderma reesei* has been used as a robust chassis for ophiobolin F biosynthesis [59] and so on.

The well-established fermentation parameters and scale-up methods of filamentous fungal chassis systems have enabled numerous successful large-scale fermentation cases. For example, the implementation of aerated and agitated submerged fermentation with *Blakeslea trispora* enabled β-carotene production reaching 30 mg/g dry cell weight (approximately 3 g/L) and established the basis for its successful industrial-scale manufacturing [60,61]. The filamentous fungus *Gibberella fujikuroi* is currently the primary strain used for the industrial production of gibberellins, owing to its capacity for high titers. This is evidenced by the work of Eleazar et al., who achieved a maximum titer of 3.9 g/L using a fluidized bed bioreactor with calcium-polygalacturonate as an immobilization carrier [62]. These successful applications collectively underscore the significant potential of filamentous fungal platforms for the synthesis and application of specialized terpenoids. This, in turn, underscores the need to develop a broader repertoire of filamentous fungal chassis to accommodate the diverse biosynthetic requirements of various terpenoid products. We have selected *B. cinerea* as the foundation for our terpene chassis. This decision was based not only on its status as a model strain, but also because we have acquired a series of patented *B. cinerea* strains that are capable of producing significant amounts of ABA, with the highest fermentation yield reaching 7 g/L [17]. This yield is the highest level of ABA yield reported in the literature. Our use of a proprietary *B. cinerea* strain allows us to have complete control over the entire process, from research to production. This level of autonomy effectively addresses common challenges in the industry, such as unstable supply chains and high costs due to strain monopolies. We have also obtained genomic and transcriptomic information of these ABA-producing strains and analyzed the synthesis and regulation mechanisms of ABA. As a sesquiterpenoid, ABA is synthesized in *B. cinerea* via the mevalonate (MVA) pathway—the central route for terpenoid backbone formation. This indicates that the native physiological framework of *B. cinerea* is inherently conducive to terpenoid production. Additionally, we have established a large-scale fermentation and extraction process for ABA production from *B. cinerea* strains, which may help us to rapidly translate newly developed terpenoid products into commercial production and application. In summary, the advantages of using *B. cinerea* as a terpenoid-producing fungal chassis strain included: (1) independent intellectual property rights, (2) clear genetic background, (3) inherent metabolic predisposition, and (4) mature scale-up production and established industrial application technologies. ABA itself is a terpene compound. This provides a solid foundation and potential for using this strain to produce other terpene compounds. While there are reports in the literature on the synthesis of terpenes using other fungi, we believe that *B. cinerea*, which is used for ABA synthesis, has the potential to synthesize other terpene compounds.

The genetic engineering of filamentous fungi for heterologous gene expression, gene complementation, overexpression, and promoter replacement has slowed due to the low efficiency of homologous recombination. While strategies such as *ku70/ku80* deletion or increasing the length of the homologous arm to increase recombination have shown promise in model systems [63,64], their application in industrial strains is limited. A commonly used technique for exogenous gene expression and endogenous gene overexpression in filamentous fungi is polyethylene glycol-mediated transformation (PMT) and ATMT [65]. However, the ATMT method involves multiple intricate steps and resistance selection and results in random genomic integration of the target gene. The random integration of transgenes can lead to significant phenotypic variation among transformants, and extensive screening is needed to eliminate position-dependent effects [65]. These technical challenges greatly hinder the iterative improvement of industrial fungal strains. In our study, we successfully achieved site-specific gene integration in the *B. cinerea* genome with high efficiency using the RNP-based CRISPR/Cas9 system. This targeted integration approach minimizes position effects, which are commonly associated with random T-DNA insertion via ATMT. Furthermore, this system allows for *in vivo* gene replacement of functionally validated targets, previously characterized by *in vitro* point mutations or truncation, enabling direct functional assessment in their native genomic contexts. Our results demonstrate that the CRISPR/Cas9 system developed in this study enables both single- and multigene editing, as well as precise targeted integration in *B. cinerea*. Importantly, the edited genetic traits remained stable across multiple generations without reverting to those of the wild type, highlighting the reversal of this system for long-term genetic manipulation and metabolic pathway engineering in this industrial-related fungal species. We will focus on expressing heterologous terpenoid synthases of interest in our engineered strain in our future work.

In conclusion, in this study, an efficient CRISPR/Cas9-based gene-editing system for the ABA-overproducing strain *B. cinerea* TB-31 was developed. The entire ABA BGC was successfully deleted, resulting in the △*bcaba1234* strain. Since *B. cinerea* is currently the only strain capable of industrial-scale sesquiterpene ABA production, the △*bcaba1234* strain is hypothesized to share favorable terpenoid synthesis traits. Multiomics analysis revealed that the △*bcaba1234* strain exhibited enhanced flux toward terpenoid precursors, rendering it a more versatile chassis for the biosynthesis of other terpenoids. Future work will utilize this CRISPR/Cas9 platform to integrate high-value genes or gene clusters into the △*bcaba1234* strain. With optimized cultivation conditions and scaled fermentation processes, this engineered strain is expected to greatly facilitate industrial-scale production of fungal-derived terpenoids, accelerating their commercial synthesis and applications.

## CRediT authorship contribution statement

**Xiao-Nan Hou:** Writing – review & editing, Writing – original draft, Visualization, Validation, Software, Methodology, Investigation, Formal analysis, Data curation. **Dan Shu:** Writing – review & editing, Writing – original draft, Supervision, Investigation, Funding acquisition, Conceptualization. **Tian-Fu Li:** Writing – review & editing. **Qi Yang:** Writing – review & editing. **Zhe-Min Li:** Writing – review & editing. **Di Luo:** Writing – review & editing. **Jie Yang:** Writing – review & editing. **Zhi-Ying Yan:** Writing – review & editing. **Hong Tan:** Writing – review & editing.

## Funding

This study was funded by the 10.13039/501100001809National Natural Science Foundation of China (32070059, 31501005) and Key Projects of 10.13039/501100012452Chengdu Institute of Biology, Chinese Academy of Sciences (CIBGG2025) and supported by Chengdu Institute of Biology, Chinese Academy of Sciences, Grant NO. E5S312. The funding agencies did not partake in designing the study, collecting, analyzing and interpreting the data or writing the manuscript.

## Declaration of competing interest

The authors declare that they have no known competing financial interests or personal relationships that could have appeared to influence the work reported in this paper.
