# Supplementary material for: Metabolic reprogramming of abscisic acid-producing strain Botrytis cinerea TB-31 toward terpenoid biosynthesis using a CRISPR/Cas9 ribonucleoprotein system
Source: Synth Syst Biotechnol. 2025 Dec 31;12:238–54. doi: 10.1016/j.synbio.2025.12.002 (PMC12804166; doi:10.1016/j.synbio.2025.12.002)
Supplement: Multimedia component 1 [file mmc1.docx]

**Supplementary Figures**


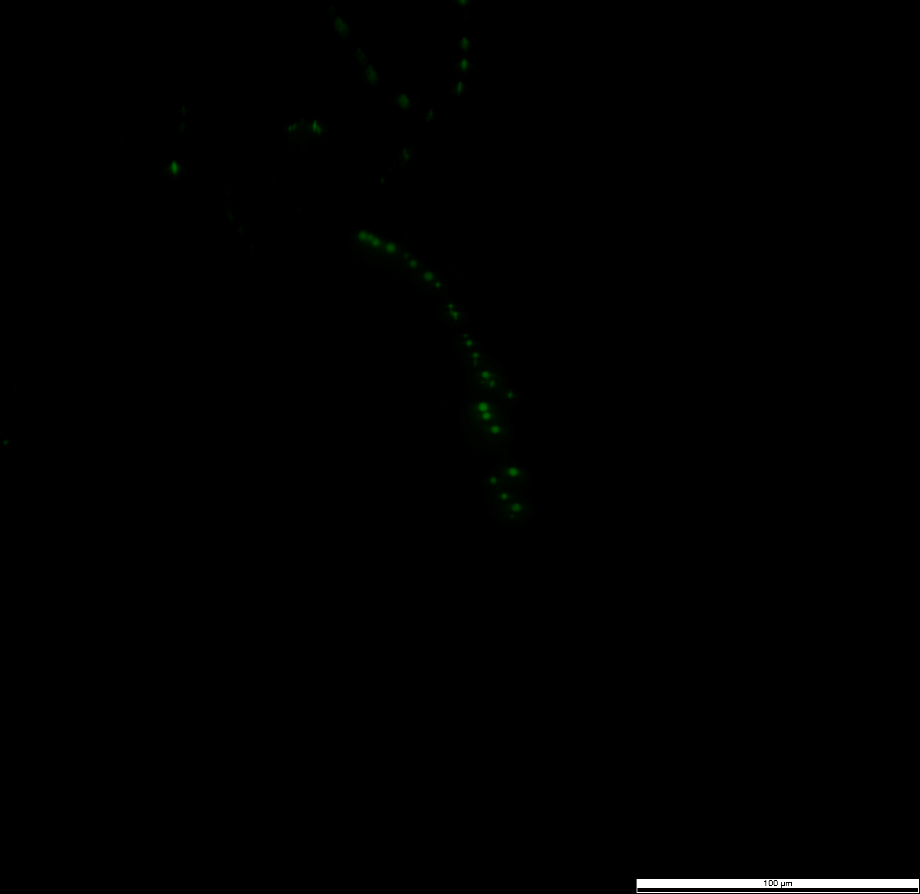

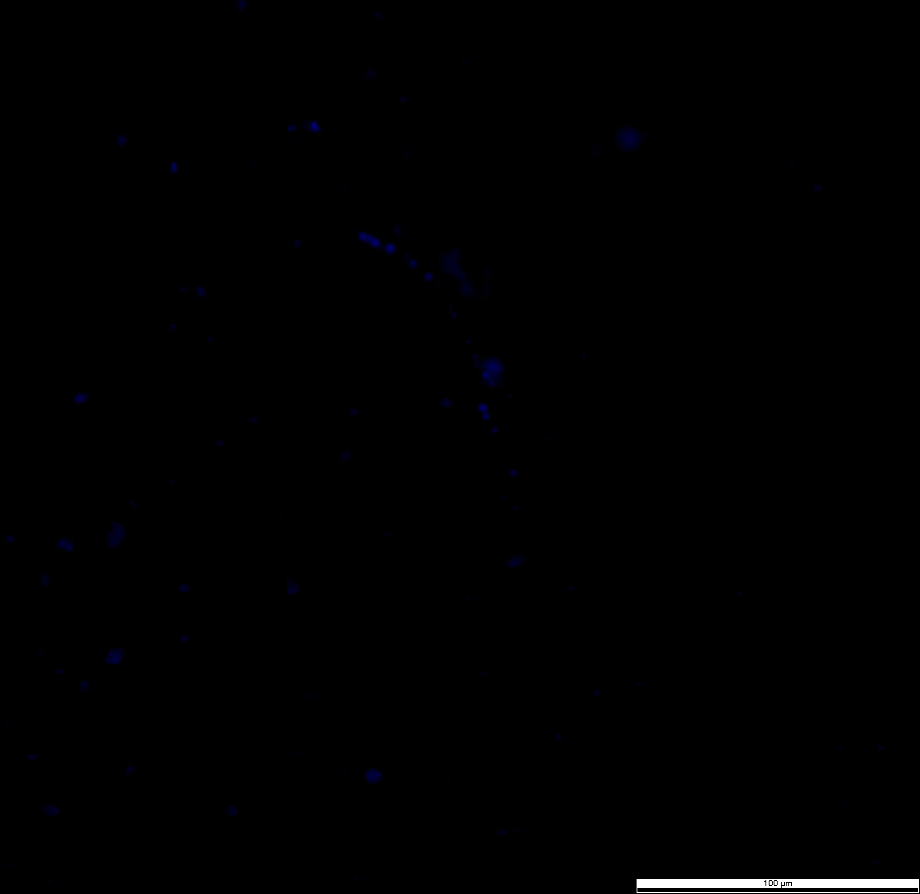

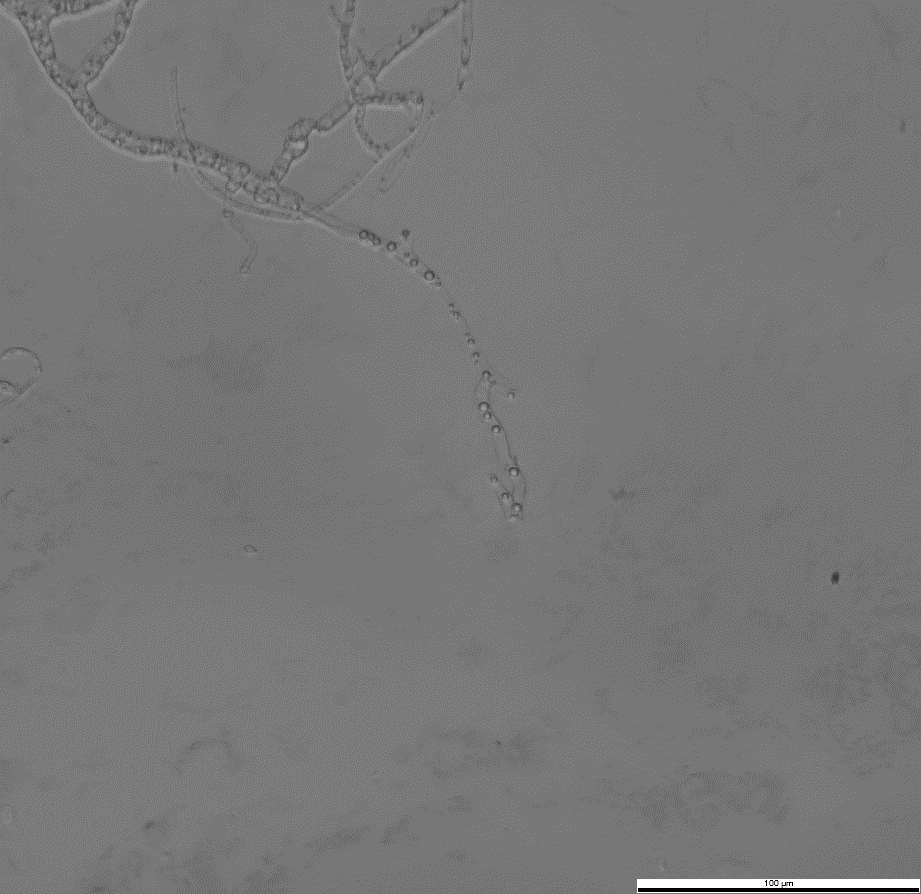

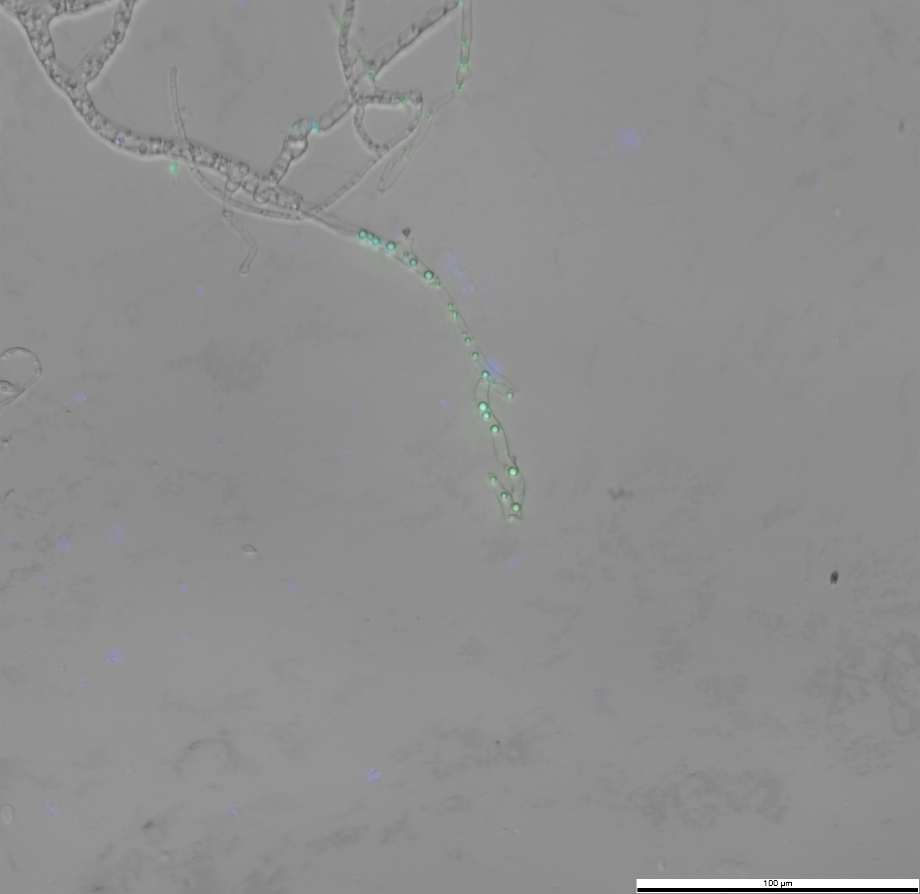


Light GFP DAPI Merge

**Fig. S1** Successful nuclear localization of EGFP mediated by tandem fusion of 4NLSs. The EGFP was fused with four tandem NLS sequences, enabling efficient targeting to the nucleus. Fluorescence microscopy images show the nuclear localization of EGFP in 3 days old germlings on glass slides, with nuclei stained with DPAI (blue). Scale bar: 25 µm.

**
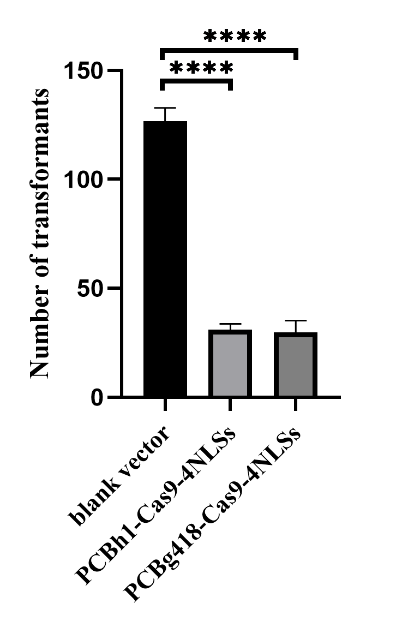

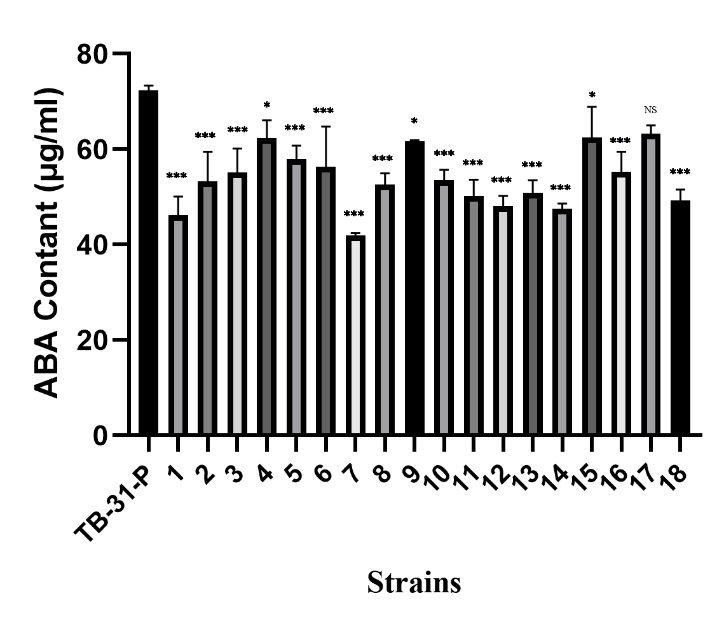
**

**Fig. S2** (A)Transformants with random genomic integration of *Cas9-4NLSs*. The *Cas9-4NLSs* coding sequence was cloned into pcbh1 and pcbg418 to yield the pCBh1-*Cas9-4NLSs* or pcbg418-*Cas9-4NLSs* plasmids, after which both the recombinant plasmid and empty vector control were transformed into TB-31 using *Agrobacterium tumefaciens*-mediated transformation (ATMT). Data were presented as means ± SEM from three independent biological replicates. Statistical significance was determined using one-way ANOVA. *, *P* < 0.05; ***, *P* < 0.001. (B) Integrated expression of Cas9-4NLSs alters ABA content in transformants. ABA content was quantified and compared in 18 randomly selected transformants with genomically integrated Cas9-4NLSs and the control strain TB-31-P (TB-31transformed with the empty vector pCBh1-blank). Most transformants (17 out of 18) expressing Cas9-4NLSs showed a significant decrease in ABA levels as measured by HPLC. Data were presented as means ± SEM from three independent biological replicates. Statistical significance was determined using one-way ANOVA. *, *P* < 0.05; ***, *P* < 0.001.

B

A


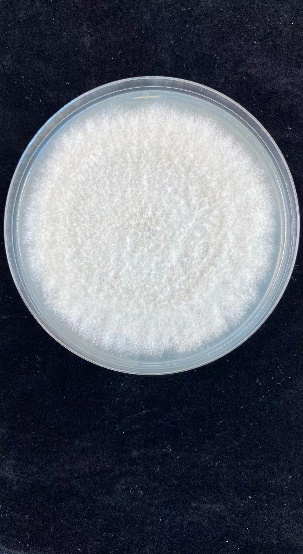

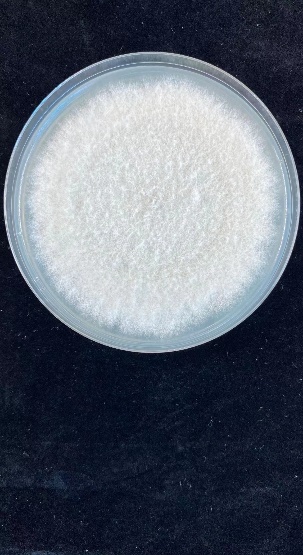

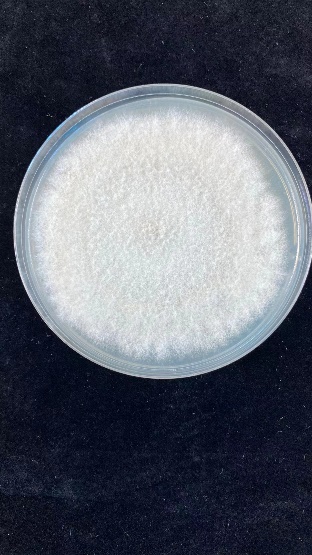

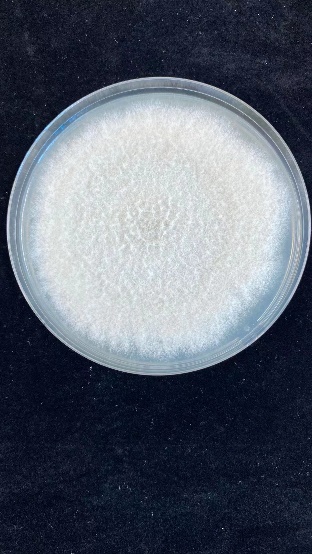


TB-31 pCBg418-*Cas9*-*4NLSs* pCBh1-*Cas9*-*4NLSs* pCBh1-*Cas9*-*EGFP*-*4NLSs*

**Fig. S3** Integrated expression of *Cas9-4NLSs* shows no significant impact on colonial morphology. The growth of *B. cinerea*strains with *Cas9-4NLSs* integrated into the genome was compared with wild-type TB-31 on PDA plates. After 7 days of incubation, no significant differences in colony size, morphology, or growth rate were observed.


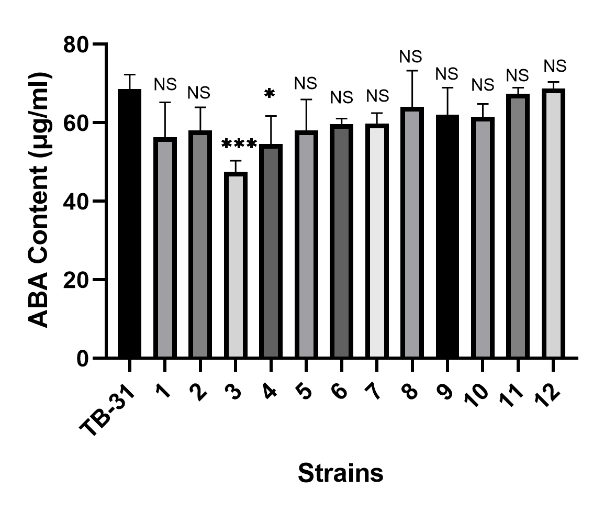


**Fig. S4** The transient expression of Cas9-4NLSs did not significantly affect ABA production in most strains. Data were presented as means ± SEM from three independent biological replicates. Statistical significance was determined using one-way ANOVA. *, *P* < 0.05; ***, *P* < 0.001.


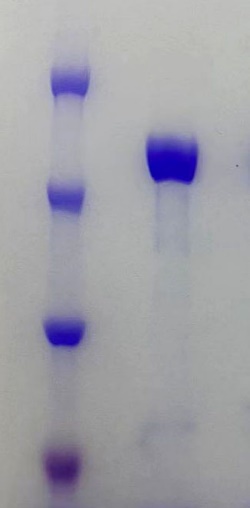


kDa M E

190-

140-

95-

70-

-Cas9

**Fig. S5** SDS-PAGE analysis of purified Cas9 protein. Purified Cas9 protein was separated by 10% SDS-PAGE and visualized by Coomassie Brilliant Blue staining. The gel shows a single band at the expected molecular weight of 164 kDa, indicating high purity of the Cas9 protein. Lane M: Prestained Protein marker (Blue Plus V, 10-190 kDa); Lane E: Purified Cas9 protein.

**
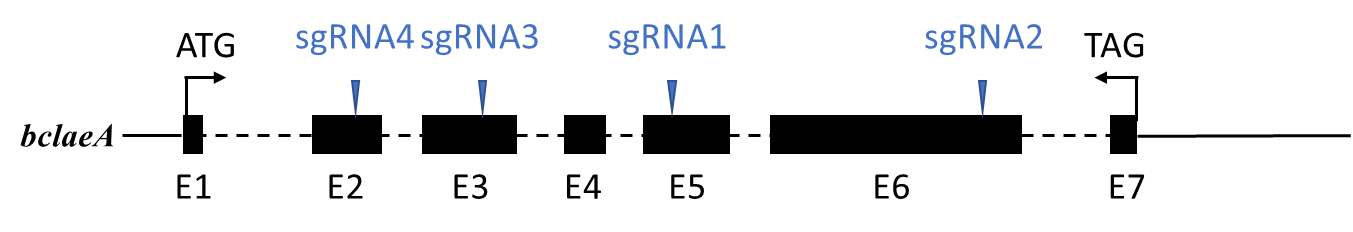
**

**Fig. S6** The sgRNA target sites of *bclaeA*. The schematic diagram illustrates the genomic structure of *bclaeA*, with exons (E) represented by solid boxes, 5′ and 3′ UTRs by solid lines, and introns by dashed lines. The sgRNA target sites are indicated by inverted triangles () in blue.


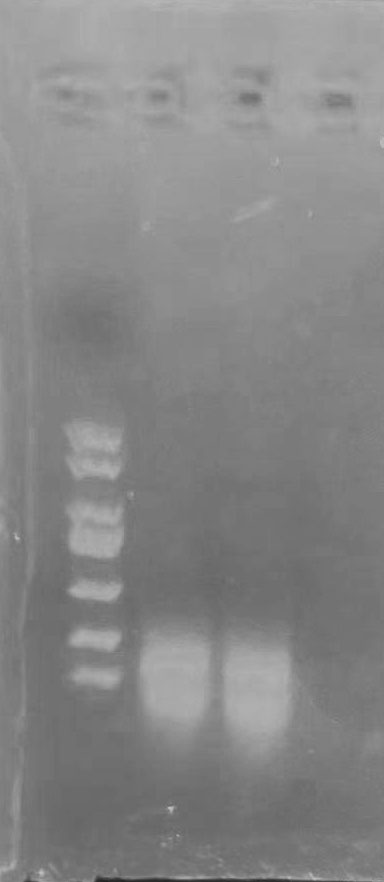


bp M 1 2

-200 nt

2000-

1500-

1000-

750-

500-

250-

100-

**Fig. S7** Agarose gel electrophoresis analysis of *in vitro* transcribed single guide RNAs (sgRNAs). *In vitro* transcribed sgRNAs were separated on a 2% agarose gel and visualized under UV light. Lane M: DNA molecular weight marker; Lane 1~2: *In vitro* transcribed sgRNAs.


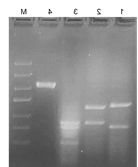


bp M 1 2 3 4

5000-

3000-

2000-

1500-

1000-

750-

500-

**Fig. S8** *In vitro* cleavage activity assay of Cas9-sgRNA RNP complexes. The cleavage activity of Cas9-sgRNA RNPs was assessed by incubating Cas9 protein (6 µg) with different sgRNAs targeting*bclaeA* gene, followed by agarose gel electrophoresis. Lane M: 5000 bp DNA marker; Lane 1: Control (uncut *bclaeA* 2198 bp); Lane 2: Cas9 + *bclaeA*-sgRNA-1 and *bclaeA-*sgRNA-2 (882 bp+796 bp+520 bp); Lane 3: Cas9 + *bclaeA-*sgRNA-1 (1316 bp + 882 bp); Lane 4: Cas9 + *bclaeA-*sgRNA-2 (1402 bp+796 bp). The gel shows distinct cleavage products, confirming the activity of the Cas9-sgRNA complexes.


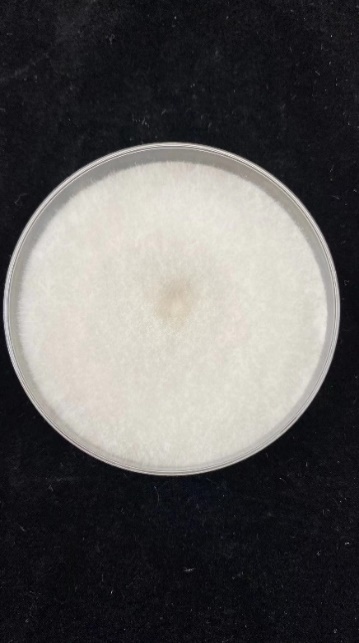

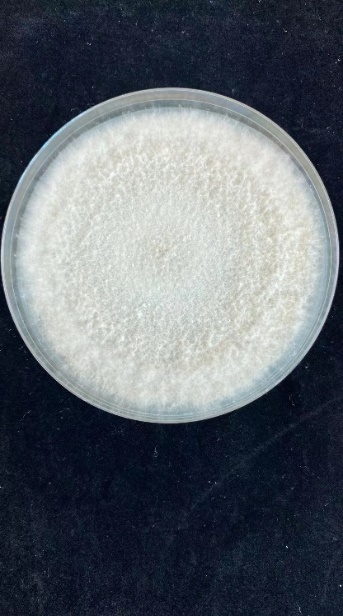


TB-31 △*bclaeA*

**Fig. S9** Colonies formed by *B. cinerea* TB-31 and △*bclaeA* strains on PDA plates 7 days after inoculation.


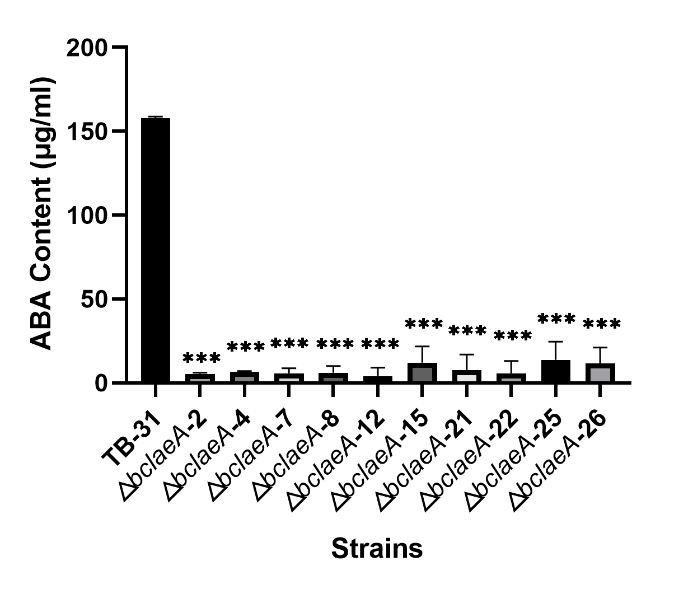


**Fig. S10** ABA content in the △*bclaeA* strains. The ABA content in the △*bclaeA* strains was quantified using HPLC and compared with the wild-type strain. Data are presented as means ± SEM from three independent biological replicates. Statistical significance was determined using one-way ANOVA. ***, *P* < 0.001. The results indicate a significant decrease in ABA levels in the △*bclaeA* strains and the success of gene knockout was confirmed.

**
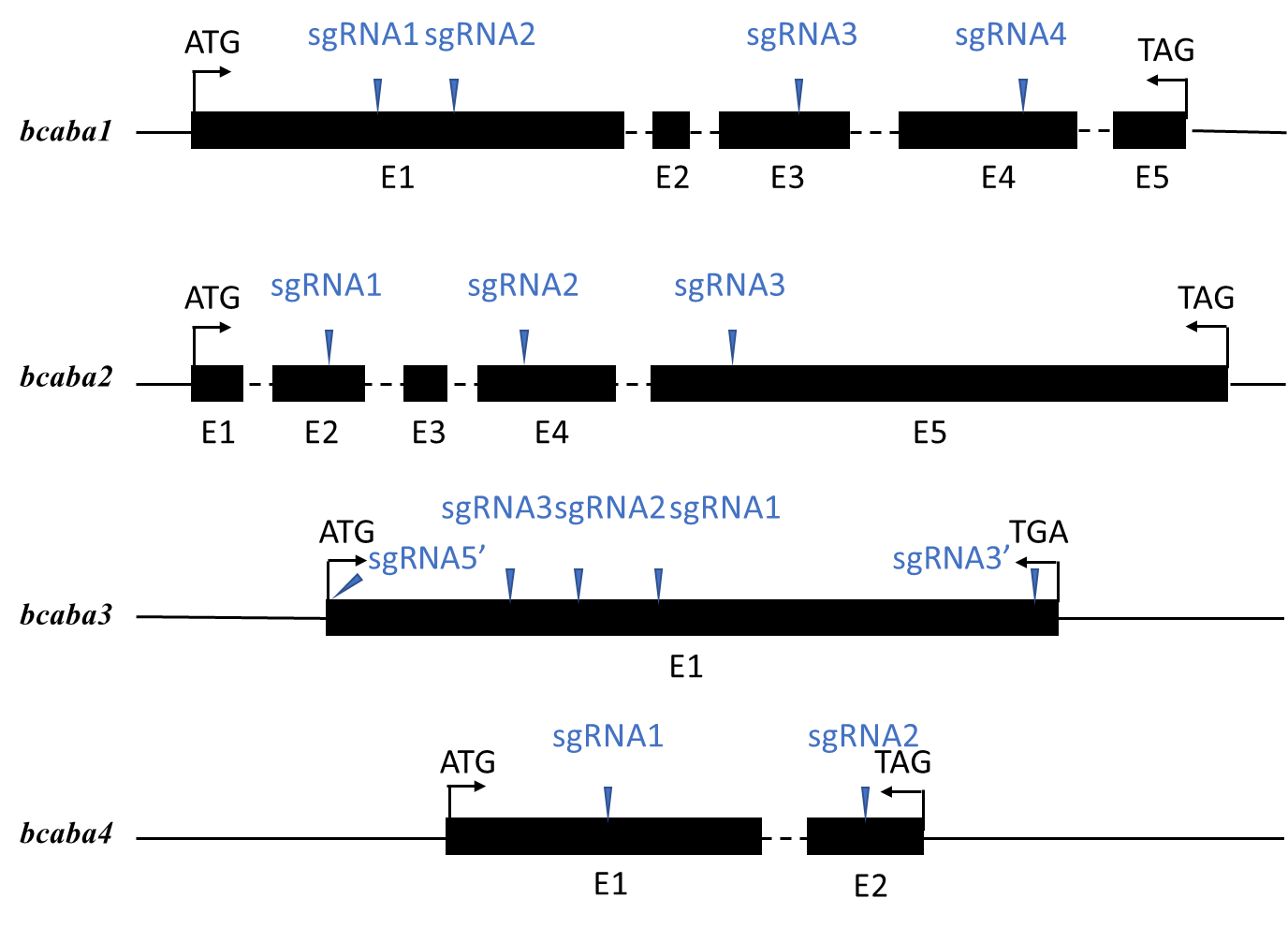
**

**Fig. S11** sgRNA target sites in *bcaba1*, *bcaba2*, *bcaba3* and *bcaba4*. The schematic diagram illustrates the genomic structure of *bcaba1, bcaba2, bcaba3 and bcaba4.* with exons (E) represented by solid boxes, 5′ and 3′ UTRs by solid lines, and introns by dashed lines. The sgRNA target sites are indicated by inverted triangles () in blue.

bp M 1 2 3 4

bp M 1 2 3 4 5

B

A


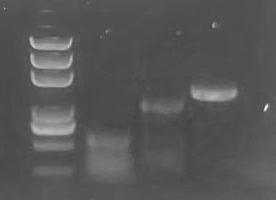

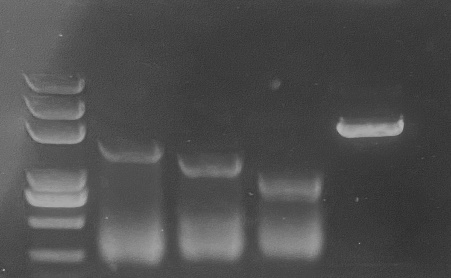
**
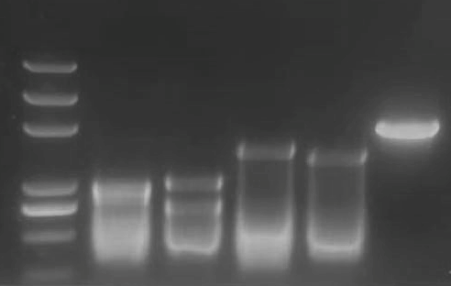
**
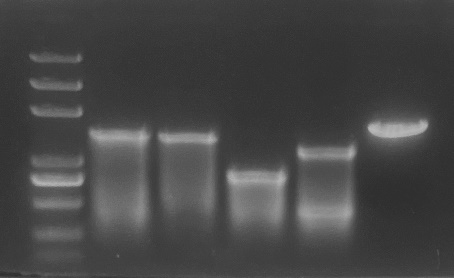


**Fig. S12** *In vitro* cleavage activity assay of different sgRNAs targeting *bcaba1/2/3/4*. The cleavage activity of Cas9-sgRNA ribonucleoproteins (RNPs) was assessed by incubating 6 µg of Cas9 protein with sgRNAs targeting the *bcaba1*, *bcaba2*, *bcaba3*, or *bcaba4* genes, followed by incubation with pre-amplified genomic DNA fragments and analysis via agarose gel electrophoresis. (A) Lane 1: Cas9 + *bcaba1*-sgRNA-1 (1533 bp+259 bp); Lane 2: Cas9 + *bcaba1*-sgRNA-2 (1445 bp+347 bp); Lane 3: Cas9 + *bcaba1*-sgRNA-3 (989 bp + 803 bp); Lane 4: Cas9 + *bcaba1*-sgRNA-4 (1369 bp+423 bp); Lane 5: uncut gDNA *bcaba1* 1792 bp. (B) Lane 1: Cas9 + *bcaba2*-sgRNA-1 (1550 bp+260 bp); Lane 2: Cas9 + *bcaba2*-sgRNA-2 (1255 bp+555 bp); Lane 3: Cas9 + *bcaba2*-sgRNA-3 (964 bp + 846 bp); Lane 4: uncut gDNA *bcaba2* 1810 bp. (C) Lane 1: Cas9 + *bcaba3*-sgRNA-1 (935 bp+300 bp); Lane 2: Cas9 + *bcaba3*-sgRNA-2 (1025 bp+511 bp); Lane 3: Cas9 + *bcaba3*-sgRNA-5′(1546 bp + 300 bp); Lane４: Cas9 + *bcaba3*-sgRNA-3′(1546 bp + 300 bp); Lane 5: uncut *bcaba3* gene flanked by 300 bp of genomic sequence (1846 bp). (D) Lane 1: Cas9 + *bcaba4*-sgRNA-1 (683 bp+608 bp); Lane 2: Cas9 + *bcaba4*-sgRNA-2 (1156 bp+135 bp); Lane 3: uncut *bcaba4* gene flanked by genomic sequence (1291 bp).

250-

500-

750-

1000-

2000-

3000-

5000-

bp M 1 2 3

250-

500-

750-

1000-

2000-

5000-

3000-

bp M 1 2 3 4 5

D

C

500-

250-

750-

1000-

2000-

3000-

5000-

100-

250-

500-

750-

1000-

2000-

3000-

5000-

**Fig. S13** PCR verification of the modified *B. cinerea* TB-31 strains. (A)~(F) Genomic DNA from wild-type (WT) and knockout strains was amplified using primers flanking the target deletion sites. The sizes of the PCR products are consistent with the predicted sizes. (A) WT (1769 bp), △*bcaba1* (1369 bp). (B) WT (1810 bp), △*bcaba2* (1381 bp). (C) WT (2880 bp), △*bcaba3* (2352 bp). (D) WT (1291 bp), △*bcaba4* (1800 bp). (E) WT (0 bp), △*bcaba124* (2354 bp). (F) WT (0 bp), △*bcaba1234* (2354 bp). M: 5000 bp DNA marker.

C

B

A

F

E

D


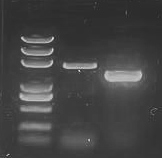


bp M WT △*bcaba1*

5000-

3000-

2000-

1000-

750-

500-

250-

100-


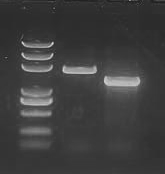


5000-

3000-

2000-

1000-

750-

500-

bp M WT △*bcaba2*

250-

100-


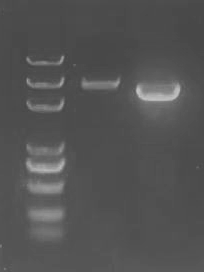


bp M WT △*bcaba3*

5000-

3000-

2000-

1000-

750-

500-

250-

100-


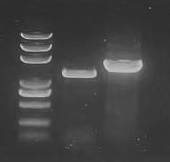


bp M WT △*bcaba4*

5000-

3000-

2000-

1000-

750-

500-

250-

100-


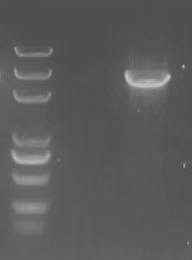


bp M WT △*bcaba124*

5000-

3000-

2000-

1000-

750-

500-

250-

100-


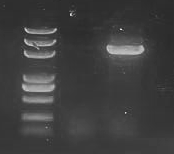


bp M WT △*bcaba1234*

5000-

3000-

2000-

1000-

750-

500-

250-

100-


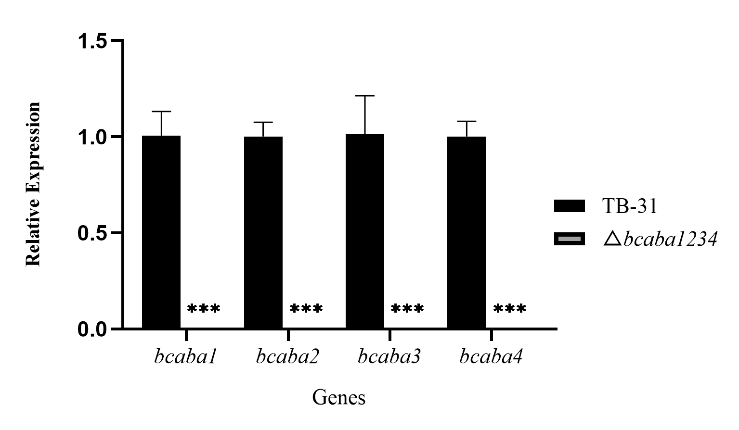

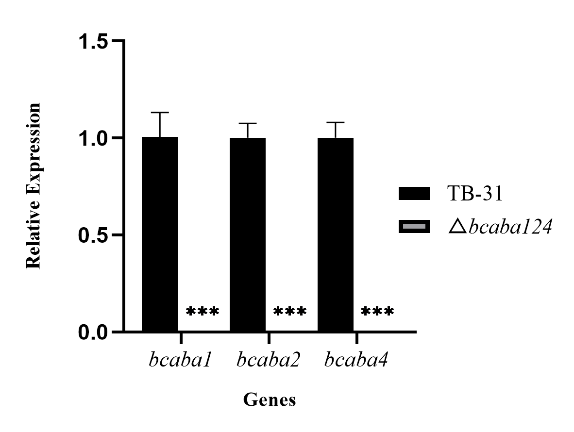


**Fig. S14** RT-qPCR verification of the modified *B. cinerea* TB-31 strains. (A)~(D) RT-qPCR validation of gene knockout in single-gene editing strains. Relative transcription levels of *bcaba1*, *bcaba2*, *bcaba3*, and *bcaba4* in wild-type (WT) and knockout (KO) strains, normalized to the *tubulin* reference gene at 6 days. Data represent mean ± SD (n = 3). Statistical comparisons were done with Student's t test. ***, *P* < 0.001. (E)~(F) Two multi-gene knockout strains (△*bcaba124* and △*bcaba1234*) showing complete loss of target gene transcription.

E

F

D

C

B

A


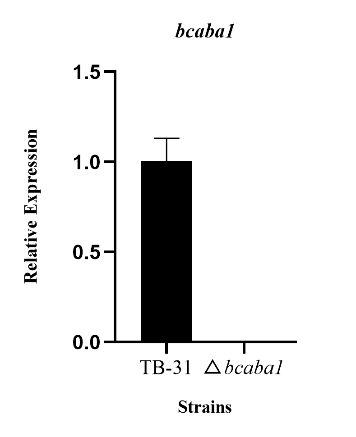

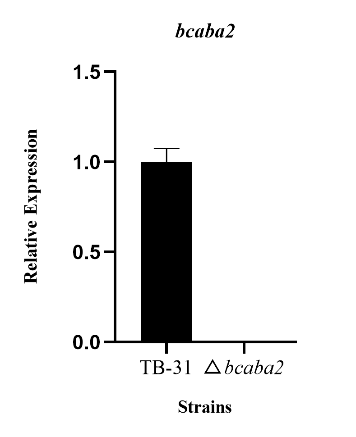

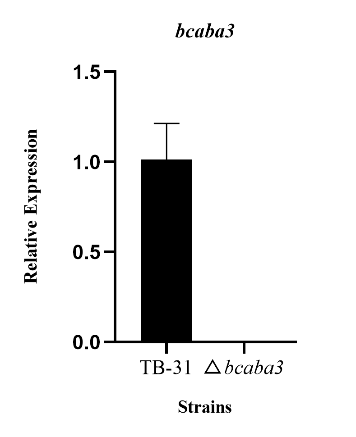

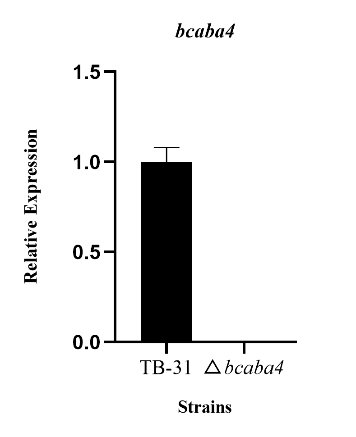


**Fig. S15** Ultra-performance liquid chromatography coupled with mass spectrometry (UPLC-MS) analysis of metabolites from TB-31, △*bcaba1,* △*bcaba2 and* △*bcaba4* strains. Total ion chromatograms (TIC) showing the overall metabolite profiles. PDA chromatograms highlighting the UV-visible absorption patterns of metabolites. (A)TB-31, (B)△*bcaba1*, (C)△*bcaba2*, (D)△*bcaba4.* The corresponding ABA precursors are indicated by arrows, respectively.

**△*bcaba1***


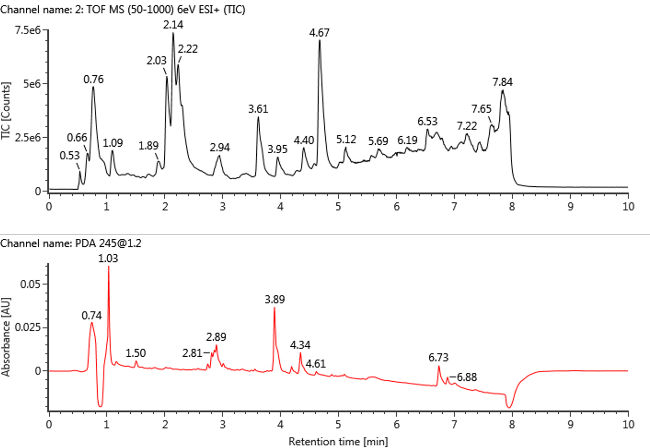


D

C

B

A

**△*bcaba2***


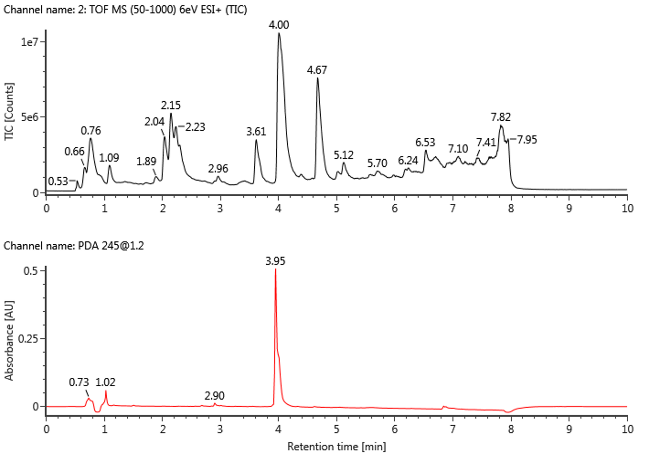


1'- deoxy-ABA

**△*bcaba4***


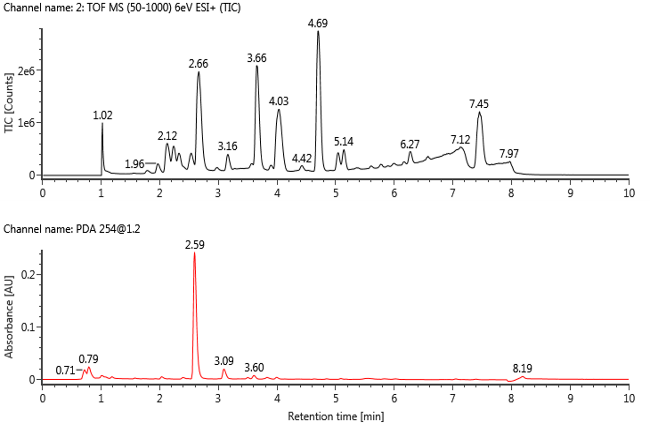


1', 4'-trans-ABA diol

**TB-31**


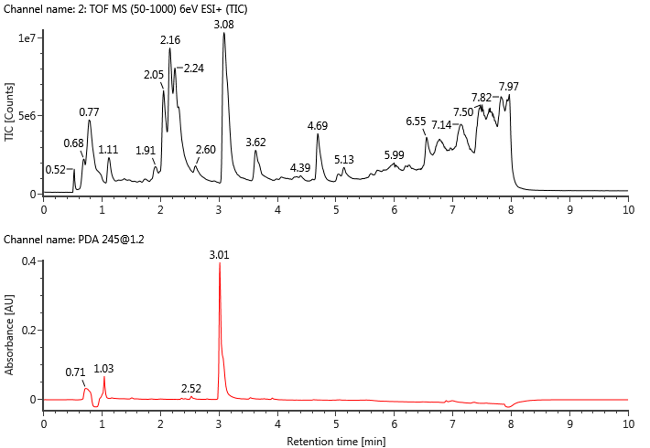


ABA

A


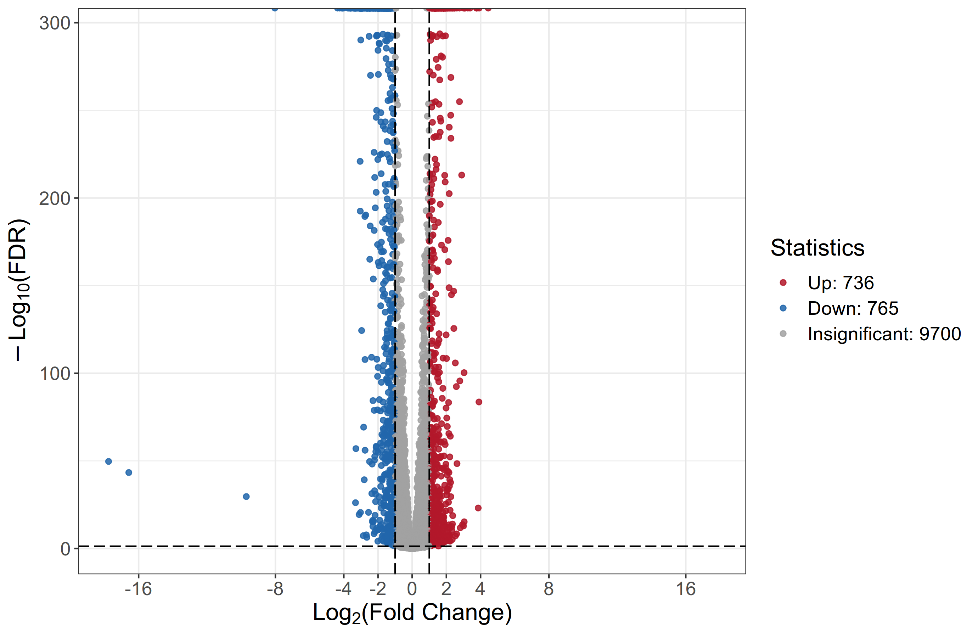
**
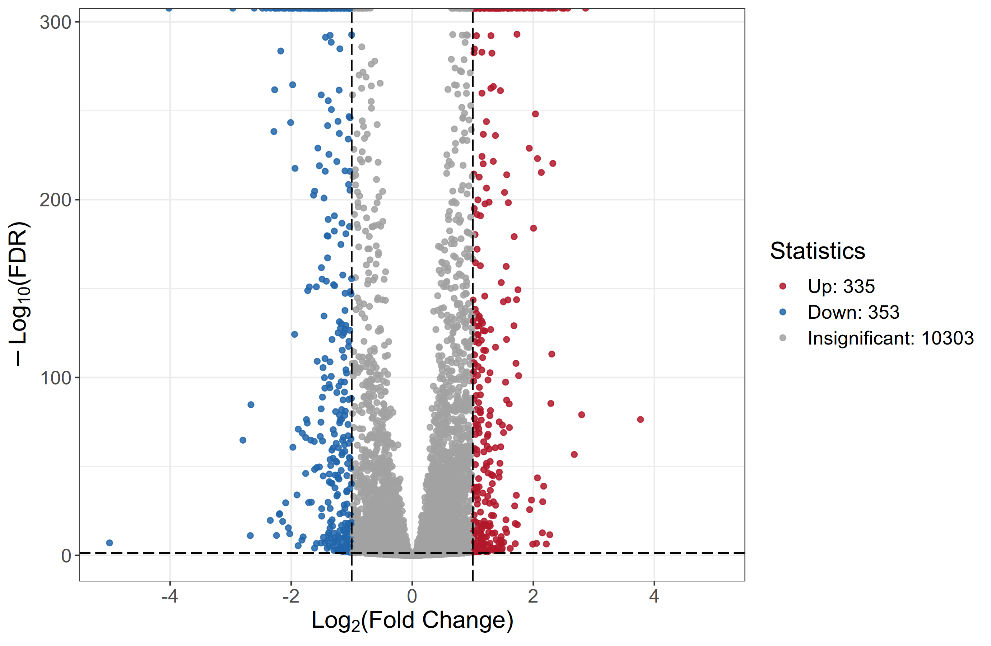
**

**Fig. S16** Volcano plot of differentially expressed genes in △*bcaba3* vs. TB-31 (A) and △*bcaba1234* vs. TB-31 (B). X-axis: log₂ fold change; Y-axis: -log₁₀(p-value). Red, blue, and gray dots denote up-regulated, down-regulated, and non-significant genes, respectively.

B

△*bcaba1234*_VS_ TB-31

△*bcaba3*_VS_ TB-31

**Fig. S17** (A-C) Volcano plot of differential metabolites in △*bcaba3* vs. TB-31(A), △*bcaba1234* vs. TB-31 (B) and △*bcaba1234* vs. △*bcaba3* (C). Red and green points denote significantly upregulated and downregulated metabolites (VIP > 1, fold change ≥ 2 and fold change ≤ 0.5, *P* < 0.05), respectively; gray points indicate non-significant metabolites.


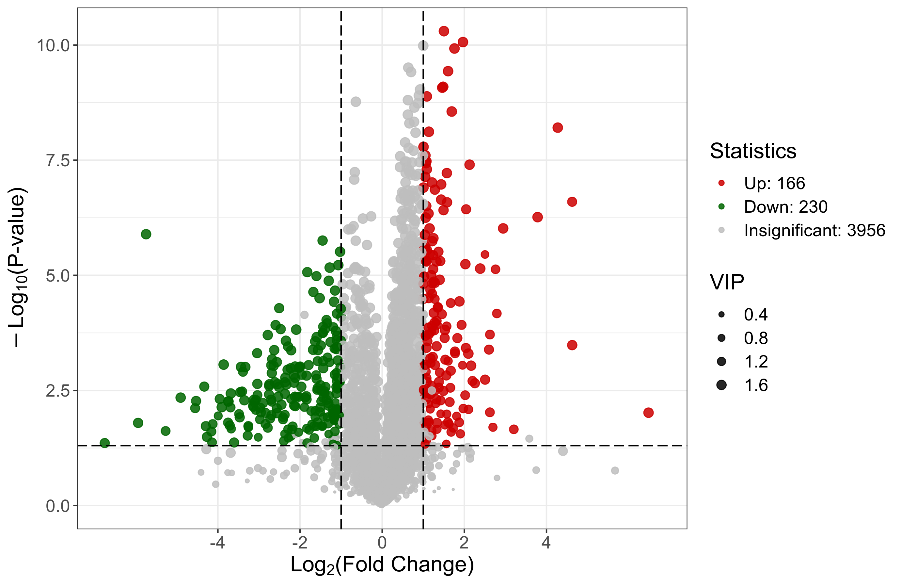


**△*bcaba1234*_VS_ △*bcaba3***


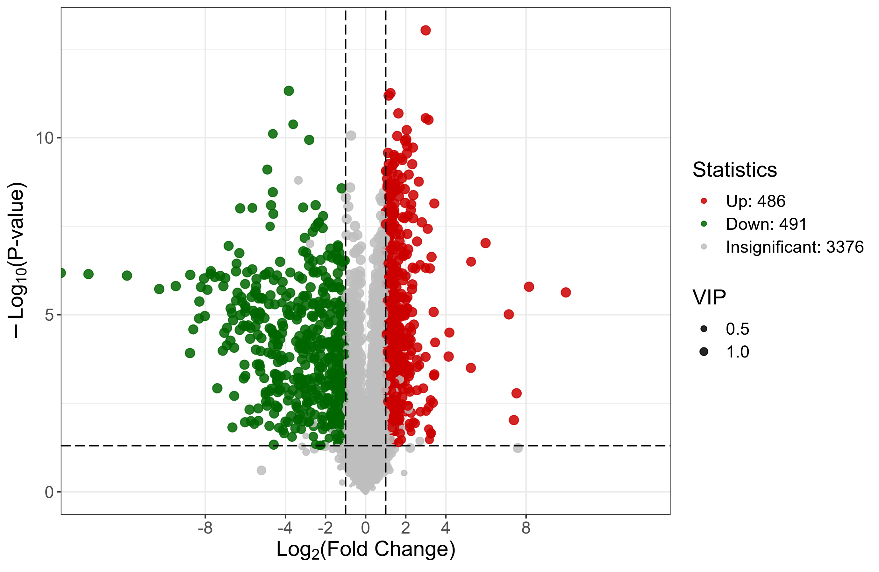


**△*bcaba1234*_VS_ TB-31**


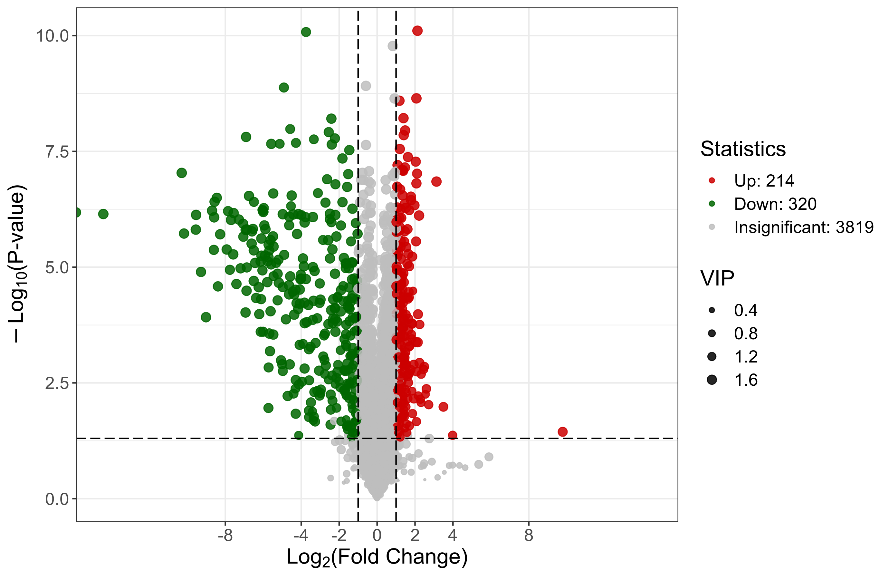


**△*bcaba3*_VS_ TB-31**

C

B

A


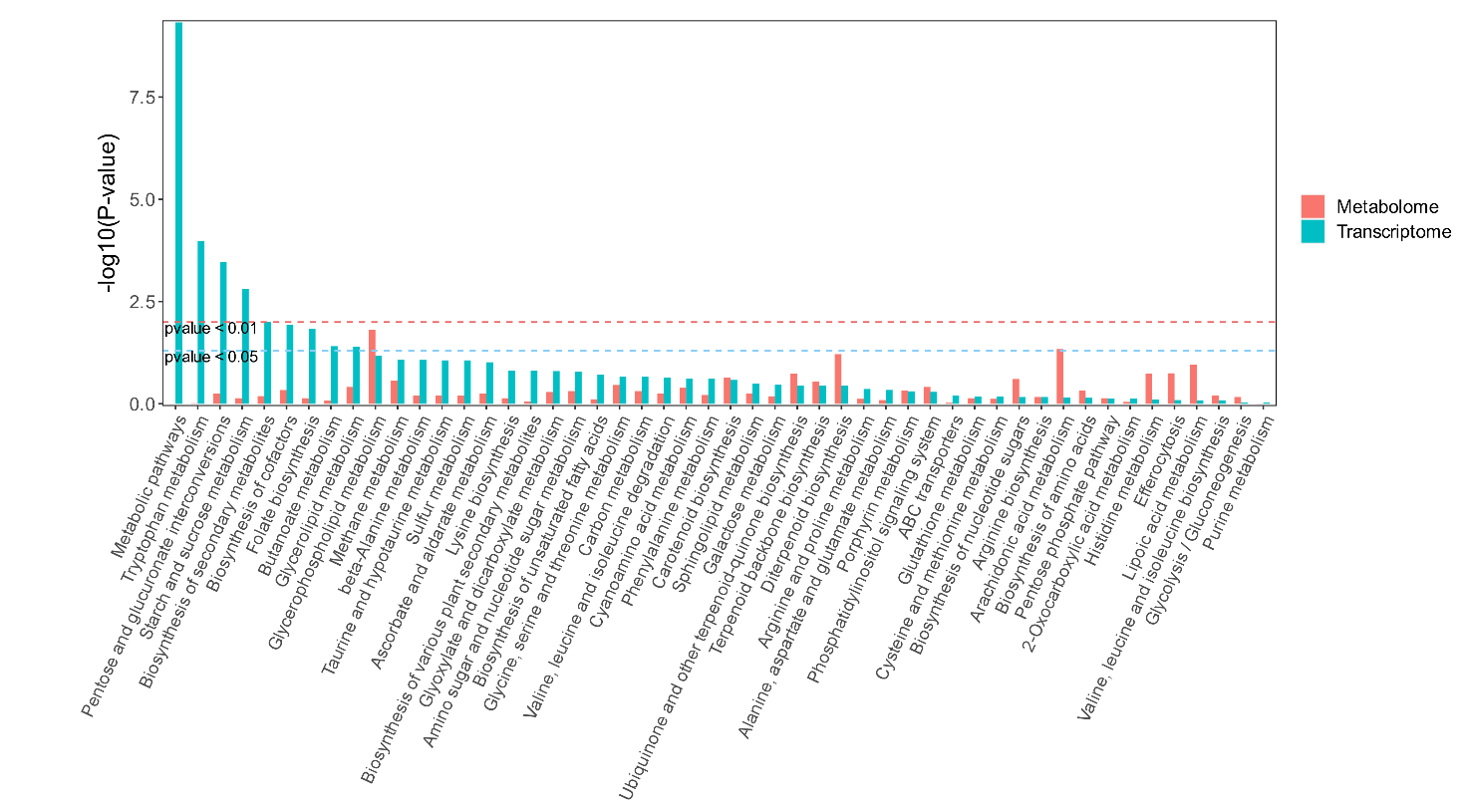


**Fig. S18** Comparative enrichment of KEGG pathways detected in both transcriptomic and metabolomic profiling in △*bcaba1234* vs. TB-31. X-axis: KEGG pathway names; Y-axis: -log10(p-value) of pathway enrichment. Metabolomic and transcriptomic results are color-coded in red and green, respectively.

**
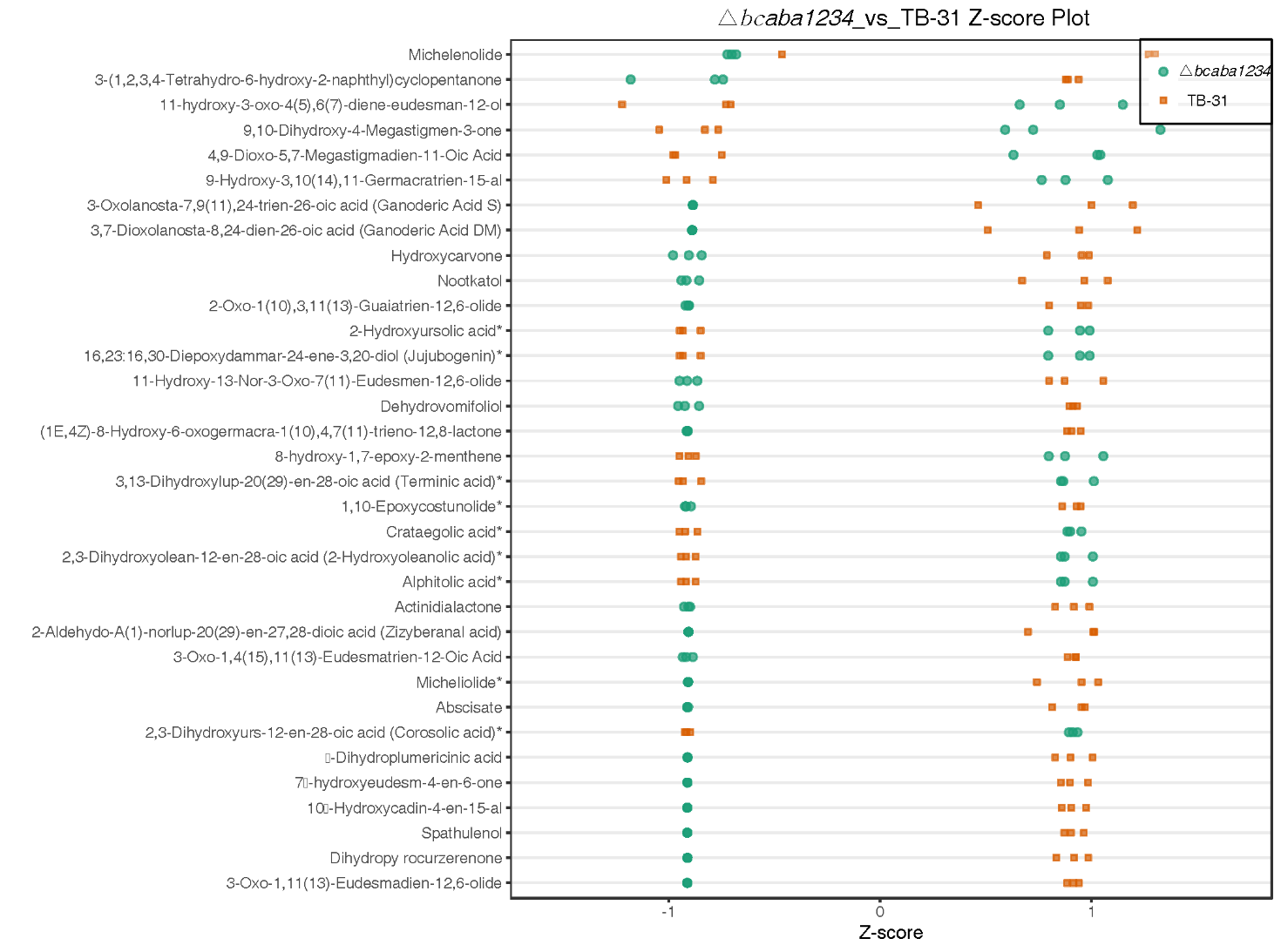
**

**Fig. S19** Z score-normalized relative abundance of differentially abundant metabolites in △*bcaba1234* vs. TB-31. Metabolites were selected based on VIP scores from PLS-DA and standardized to Z scores.

**
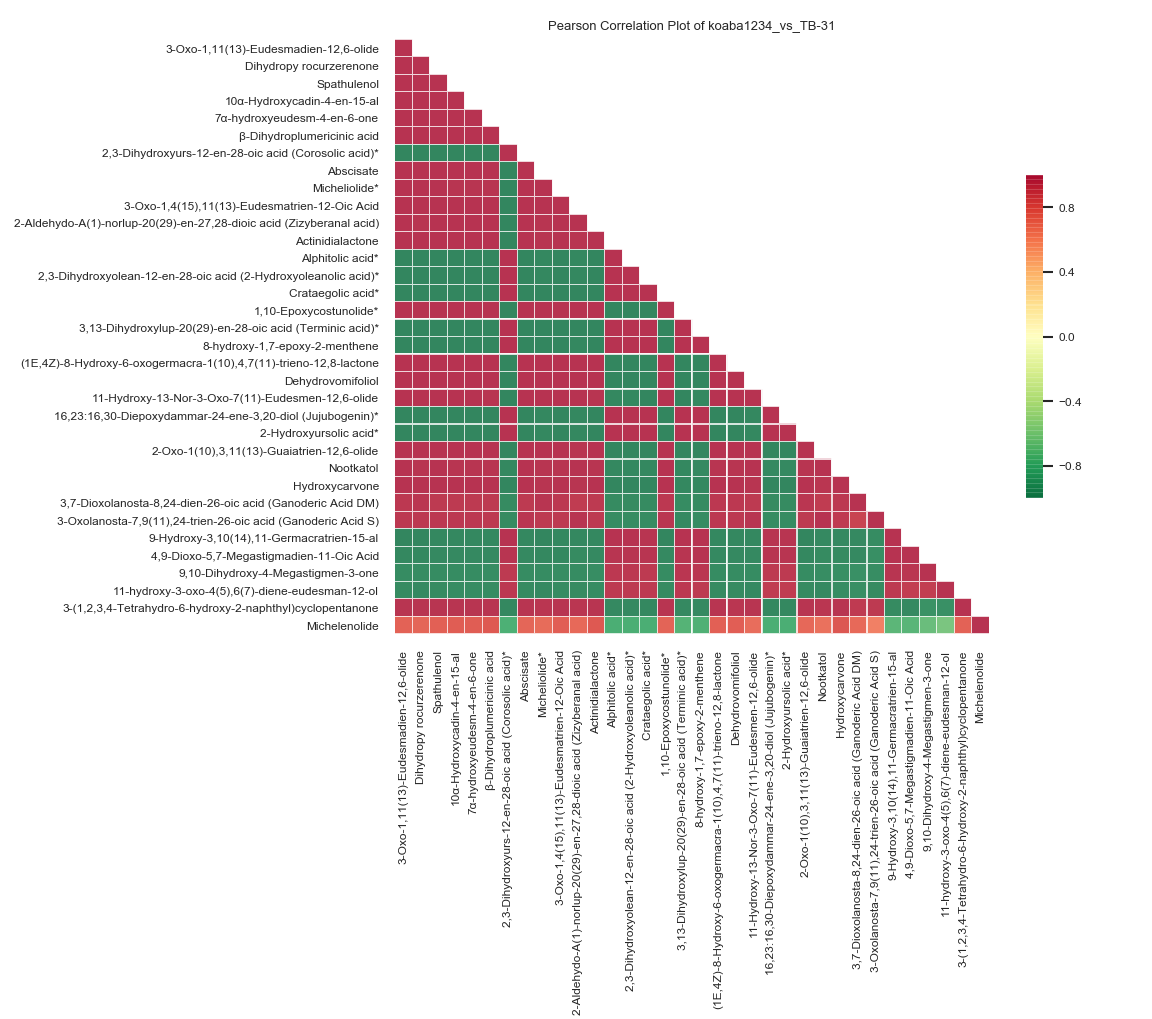
**

**Fig. S20** Correlation heatmap of differential metabolites. Pairwise Pearson correlations between differentially abundant metabolites from the △*bcaba1234* vs. TB-31 comparison. Metabolites are plotted on both axes. The color scale represents the correlation coefficient (r), with intense red and green hues indicating strong positive and negative correlations, respectively.


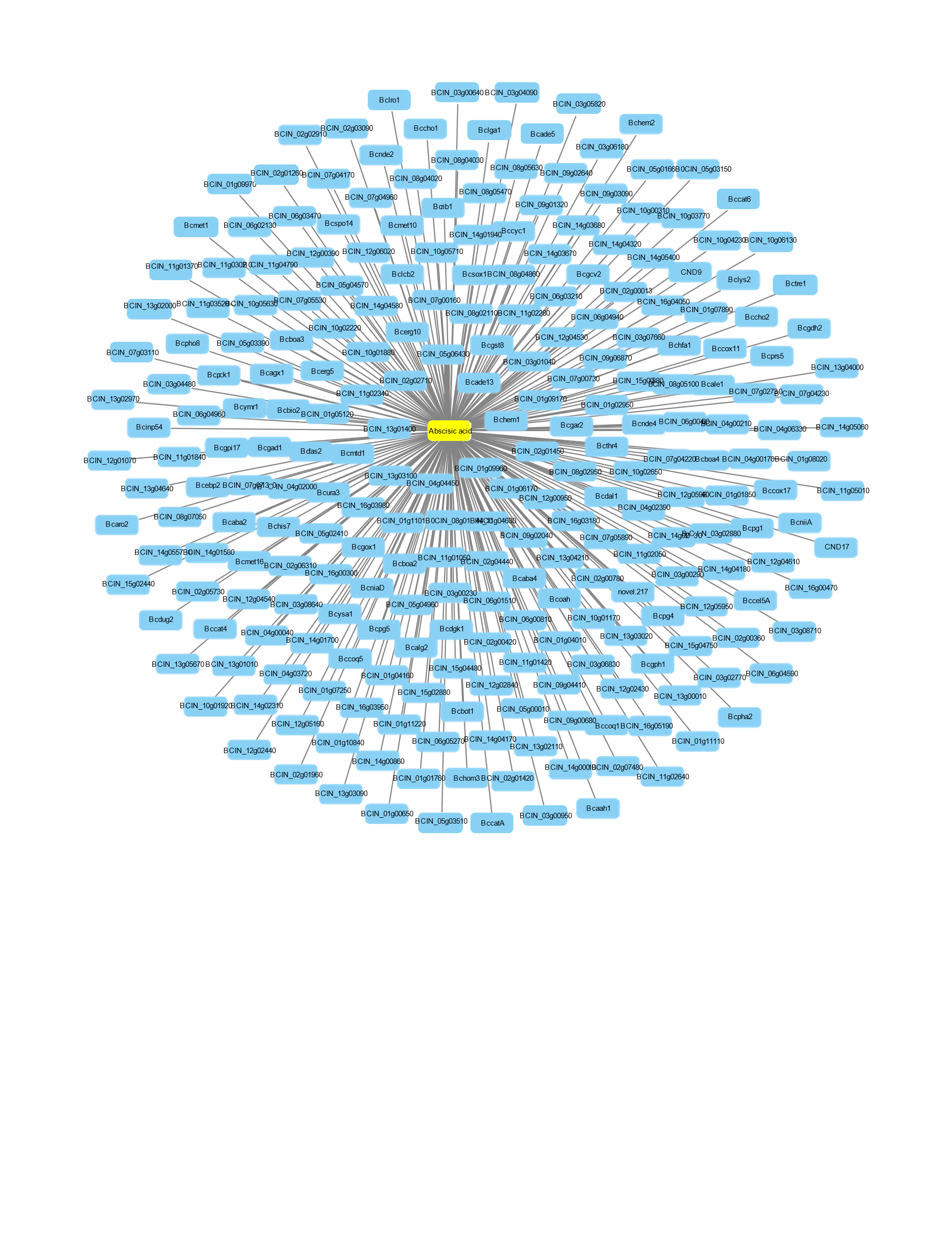


**Fig. S21** Network analysis of correlations between ABA and related genes. The network illustrates the significant pairwise correlations (|r| > 0.8, p < 0.01).


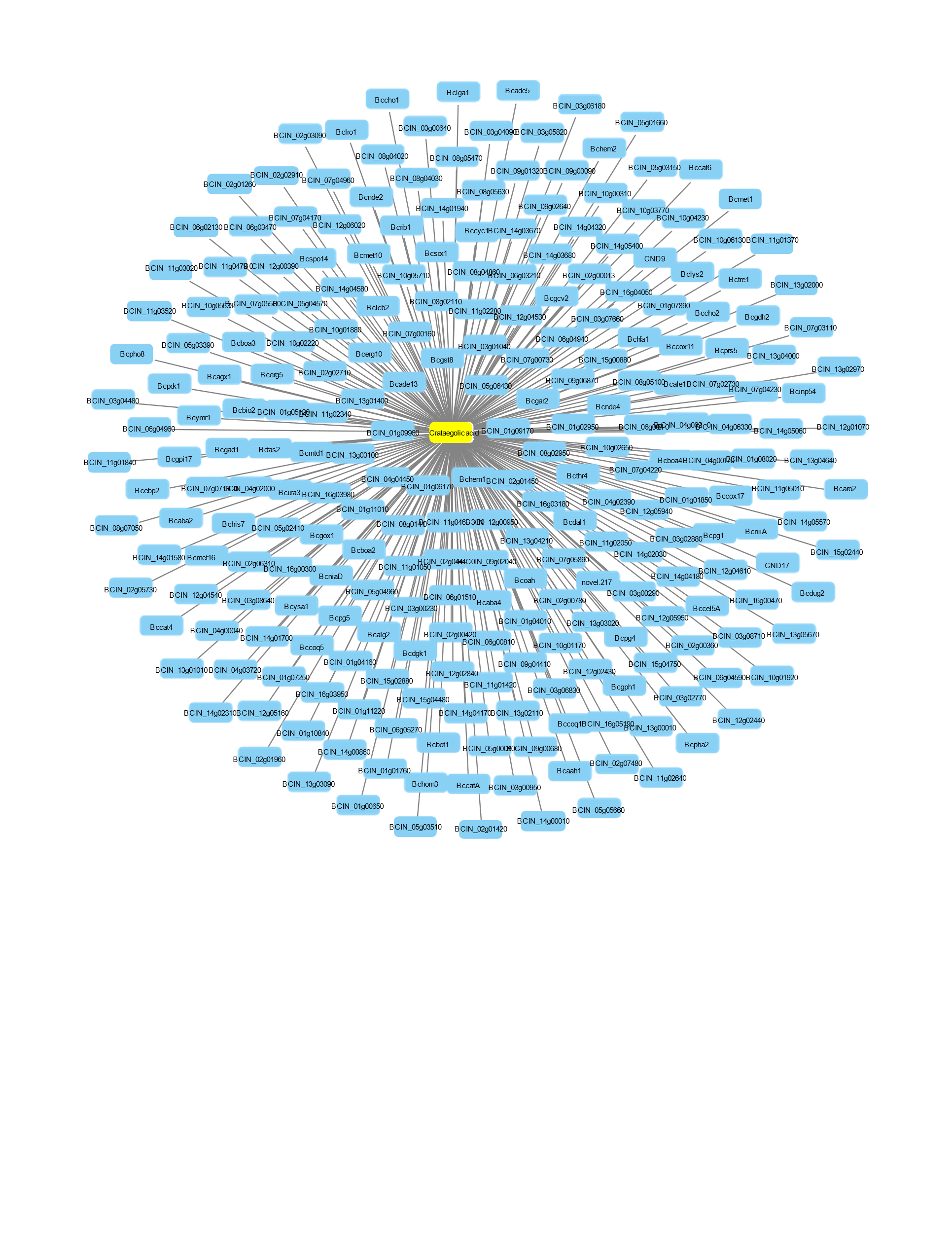


**Fig. S22** Network analysis of correlations between Stigmasterol-type triterpenoids Crataegolic acid and related genes. The network illustrates the significant pairwise correlations (|r| > 0.8, p < 0.01).
